# Time-Updated Prognostic Modeling in ICU Patients with Documented Coma or Unresponsiveness Using Routine Arterial Blood Gas Trajectories: An Exploratory Explainable Machine-Learning Study

**DOI:** 10.3390/jcm15135056

**Published:** 2026-06-29

**Authors:** Pompiliu Mircea Bogdan, Camer Salim, Roxana Elena Bogdan-Goroftei, Alina Pleșea-Condratovici, Cristian Guțu, Călin Gheorghe Buzea, Bogdan Costăchescu, Letiția Doina Duceac, Manuela Arbune, Constantin-Marinel Vlase, Irina Luciana Gurzu, Alina Mihaela Călin

**Affiliations:** 1 Doctoral School of Biomedical Sciences, Faculty of Medicine and Pharmacy, Research Center in the Medical-Pharmaceutical Field, “Dunărea de Jos” University of Galați, 800008 Galați, Romania; pompiliu.bogdan@ugal.ro; 2 Faculty of Medicine, “Ovidius” University of Constanța, 900470 Constanța, Romania; salimcamer@yahoo.com; 3 Faculty of Medicine and Pharmacy, Research Center in the Medical-Pharmaceutical Field, “Dunarea de Jos” University of Galați, 800008 Galați, Romania; cristian.gutu@ugal.ro (C.G.); letimedr@yahoo.com (L.D.D.); manuela.arbune@ugal.ro (M.A.); alina.calin@ugal.ro (A.M.C.); 4 National Institute of Research and Development for Technical Physics—IFT Iași, 700050 Iași, Romania; calinb2003@yahoo.com; 5 Clinical Emergency Hospital “Prof. Dr. Nicolae Oblu” Iași, 700309 Iași, Romania; 6 Department of Neurosurgery, “Gr. T. Popa” University of Medicine and Pharmacy, 700115 Iași, Romania; bogdan.costachescu@umfiasi.ro; 7 Emergency Military Hospital Galați Dr. Aristide Serfioti, 800150 Galați, Romania; constantin.vlase@ugal.ro; 8 Department of Preventive Medicine and Interdiciplinarity, “Gr. T. Popa” University of Medicine and Pharmacy, 700115 Iași, Romania; irina-luciana.gurzu@umfiasi.ro

**Keywords:** coma, intensive care unit, prognostication, arterial blood gas, oxygenation, physiological trajectories, ICU mortality, explainable artificial intelligence, machine learning, SHAP, decision curve analysis, cross-validation

## Abstract

**Background/Objectives:** Prognostication in ICU patients with documented coma or unresponsiveness is a high-stakes task that informs escalation of care, goals-of-care discussions, and family counselling. Conventional scores are often based on static snapshots and may not reflect early physiological evolution in heterogeneous real-world ICU populations. Routine arterial blood gases (ABG) and SpO_2_ are repeatedly measured during early ICU care and may capture clinically meaningful trajectories that can be leveraged by explainable machine learning. To develop and internally validate exploratory, time-updated explainable machine-learning models for ICU outcome in ICU patients with clinically documented coma or unresponsiveness using routine ABG/SpO_2_ measurements and physiological trajectories available at admission, 24 h, and 72 h, and to evaluate whether trajectory information adds prognostic information within a staged internal-validation framework. **Methods:** We conducted a retrospective single-centre study of 108 adult ICU patients with clinically documented coma or unresponsiveness. Predictors included demographics, comorbidity burden, COVID-19 status, baseline ABG/SpO_2_ at ICU admission, inflammatory and coagulation biomarkers, and derived ABG/SpO_2_ trajectory variables at 24 h and 72 h. Trajectory variables were defined as changes from admission to 24 h and to 72 h and were retained as missing when follow-up measurements were unavailable. The primary ICU-course outcome was ICU death versus transfer to ward. Three staged models were evaluated: Model A using baseline variables, Model B adding 24 h trajectory features, and Model C adding 72 h trajectory features. For each stage, models were analyzed with and without the derived respiratory_support index; models excluding respiratory_support were treated as the main interpretive analyses. Logistic regression, random forest, and gradient boosting (XGBoost) classifiers were assessed using repeated stratified 5-fold cross-validation with 20 repeats and aligned out-of-fold predictions. Performance was reported using AUC-ROC, precision–recall AUC, Brier score, and operating-point metrics; clinical utility was examined with decision-curve analysis. Model interpretation used SHAP and partial dependence plots. Robustness analyses included feature-exclusion sensitivity analysis for respiratory_support and a label-permutation sanity check. **Results:** ICU mortality was 65.7% (71/108). Follow-up ABG completeness was 75.9% at 24 h and 61.1% at 72 h. Because respiratory_support summarized the highest support level during the first 72 h and strongly separated outcome groups, models excluding respiratory_support were treated as the primary interpretive analyses. In the primary NoRS logistic-regression models, discrimination was moderate-to-strong, with AUC-ROC 0.822 for Model A_noRS, 0.848 for Model B_noRS, and 0.895 for Model C_noRS; bootstrap 95% confidence intervals were 0.739–0.897, 0.766–0.919, and 0.830–0.951, respectively. Measurement-availability sensitivity analyses and simple benchmark models were added to contextualize trajectory-related performance. Respiratory_support-enriched models were retained only as secondary severity-aware analyses, not as admission-only prediction models. Label permutation reduced discrimination toward chance (AUC ≈ 0.55). SHAP and partial-dependence analyses identified oxygenation variables, inflammatory burden, acid–base status, and ΔPaO_2_ at 72 h as clinically coherent contributors to predicted risk; when included, respiratory_support dominated feature attribution, consistent with its role as an organ-support intensity marker. **Conclusions:** In ICU patients with clinically documented coma or unresponsiveness, explainable machine-learning models using routine ABG/SpO_2_ trajectories within the first 72 h are feasible and may provide time-updated prognostic information, but the incremental value of trajectory-enriched models over simpler admission-only benchmarks remains unproven. Trajectory-enriched NoRS models retained meaningful discrimination after removing organ-support severity, suggesting a possible physiologically meaningful signal beyond support intensity alone, although definitive incremental value over parsimonious admission-only benchmarks was not established. These findings should be interpreted as exploratory and internally validated only; they do not establish a deployable ICU mortality score, do not demonstrate superiority over established ICU severity scores, and require external validation in larger multicentre cohorts before clinical deployment.

## 1. Introduction

### 1.1. Clinical Problem: Prognostication in ICU Patients with Documented Coma or Unresponsiveness

Coma is a common and devastating presentation in the intensive care unit (ICU), arising from diverse etiologies such as post-anoxic injury, intracerebral haemorrhage, traumatic brain injury, sepsis, metabolic derangements, or mixed causes [1]. Across these conditions, clinicians are repeatedly asked to answer the same fundamental question: “What are this patient’s chances of survival and meaningful recovery?” Early prognostication in comatose patients has profound implications for treatment escalation, allocation of scarce ICU resources, and emotionally charged discussions with families about the continuation or withdrawal of life-sustaining treatments [2,3,4,5,6]. In the present study, the term “coma” is used operationally to denote clinically documented coma or unresponsiveness in a pragmatic ICU cohort, not a strictly defined neurocritical-care coma syndrome.

Traditionally, prognostic assessment in coma has relied on a combination of neurological examination (e.g., Glasgow Coma Scale, pupillary and corneal reflexes), neurophysiological tests (EEG, evoked potentials), neuroimaging, and selected serum biomarkers [2,3,4,5,6]. Over the last decade, guideline statements have emphasized a multimodal approach and recommended delaying definitive prognostication—typically to at least 72 h after return of spontaneous circulation in post-cardiac arrest patients—to reduce the risk of a self-fulfilling prophecy in which pessimistic early assessment leads to premature withdrawal of care [2,4,5,6]. However, these recommendations primarily address specific subgroups (such as post-anoxic coma after cardiac arrest) and do not fully capture the heterogeneous reality of a general ICU, where comatose patients may have multiple overlapping insults and evolving physiological instability.

From the intensivist’s perspective, prognostication in this context is not a one-off calculation but a daily dynamic process. At the bedside, intensivists continuously integrate changes in oxygenation, ventilation, hemodynamics, inflammatory markers, and organ support into their mental model of the patient’s trajectory. The same PaO_2_ or pH value can be interpreted very differently depending on whether it represents improvement, stability, or deterioration compared to the preceding 24–72 h. This temporal dimension is largely absent from most widely used severity scores (APACHE, SAPS, SOFA), which are based on static snapshots and were not designed specifically for comatose patients [7,8,9,10]. In the present study, “comatose” refers to adult patients admitted to the ICU with clinically documented unresponsiveness requiring intensive monitoring and supportive care (operational definition provided in Section 2).

### 1.2. Why Trajectories Matter in ICU Prognostication

Recent work in critical care informatics has shown that machine-learning (ML) models can improve mortality prediction in ICU patients by leveraging large electronic health record databases and high-dimensional feature sets, including both linear models and ensemble tree methods (e.g., random forests) [11,12,13,14,15,16,17]. In particular, dynamic and explainable ML approaches that incorporate time-series data—vital signs, laboratory values, and ventilatory parameters measured repeatedly over time—have been shown to outperform static models for predicting outcomes such as 90-day mortality, ICU length of stay, and risk of clinical deterioration [10,14,15,16]. Beyond linear baselines, nonlinear ensemble learners—particularly gradient boosting machines (e.g., XGBoost/GBM)—have demonstrated strong performance in ICU risk prediction tasks and are widely used for structured clinical data [18]. These frameworks provide not only point estimates of risk but also patient-specific explanations of which variables and trajectories contribute most to a given prediction [19].

Parallel efforts in neurocritical care have applied ML to coma outcome prediction using EEG, imaging, and multimodal monitoring, particularly in post-cardiac-arrest cohorts [2,4,5,6]. While these studies highlight the potential of AI-assisted neuroprognostication, they often require advanced modalities that are not universally available, and their etiology-specific focus may limit generalizability to heterogeneous ICU coma populations. For day-to-day ICU practice, models based on routine ABG/SpO_2_ data and standard biomarkers are attractive because they use low-cost, repeatedly measured variables that clinicians already interpret during bedside reassessment. Explainability remains important in this setting because prognostic estimates must be clinically interpretable and communicable in high-stakes ICU decision-making [19,20,21].

Importantly, trajectory-based prognostication is inherently time-dependent: risk estimates updated at 24–72 h reflect not only baseline severity but also early response to treatment. Because such models are intended to support high-stakes clinical decisions, evaluation should extend beyond discrimination to clinical utility metrics such as net benefit using decision-curve analysis [22]. Therefore, when trajectory features are used, models should be explicitly framed as time-updated prognostic tools, not as admission-only predictors [20,23]. Similar staged temporal-window approaches have been used in ICU-related machine-learning studies, in which predictors are incrementally added across clinically meaningful time windows to support time-updated risk estimation [24]. In this context, the goal is not to replace clinical judgment or to propose new biological phenotypes, but to provide transparent, bedside-aligned summaries of early physiological evolution that clinicians already interpret qualitatively during daily rounds [19].

### 1.3. Gap and Rationale

Despite these advances, there remains a notable gap: few studies have examined dynamic trajectories of simple physiological variables in comatose ICU patients using interpretable ML methods, especially in realistic, single-centre cohorts where resources and monitoring capabilities may be limited [15,16,20,21]. Most existing models either (i) focus on global ICU populations without isolating comatose patients as a distinct high-risk subgroup, or (ii) rely on specialized neurodiagnostic tools that may not be available in all settings [2,4,5,6].

In everyday practice, however, intensivists frequently base their early prognostic impressions on the evolution of oxygenation and acid–base status under mechanical ventilation or high-flow oxygen, the response of inflammatory markers, and the degree of organ support required in the first 72 h of ICU stay. These variables are objective and commonly recorded in routine ICU care but are rarely synthesized systematically into prognostic models tailored to comatose patients. There is therefore a strong rationale for exploring whether trajectory-based AI models using such routine data can (a) capture clinically meaningful patterns of early improvement or deterioration, (b) provide transparent explanations that match bedside reasoning, and (c) potentially support more consistent, data-informed prognostication [14,15,16,19,20]. Accordingly, we focus on interpretable, trajectory-based prediction using routinely available ABG/SpO_2_ and admission biomarkers, designed to mirror real-world ICU reasoning in a heterogeneous comatose population and to yield explanations that are clinically intelligible at the bedside [19].

### 1.4. Objectives and Hypothesis

Building on an initial concept of early outcome prediction using longitudinal physiological and laboratory data in ICU patients with documented coma or unresponsiveness, we designed a retrospective, single-centre study of 108 adult ICU patients with clinically documented coma or unresponsiveness. Our primary objective was to:

1. Develop and internally validate exploratory, time-updated machine-learning models for ICU-course outcome, defined as survival to ward transfer versus ICU death, using routine arterial blood gas parameters, SpO_2_, and selected biomarkers measured at admission, 24 h, and 72 h.

Secondary objectives were to:

2. Compare an early severity-aware baseline model with time-updated models incorporating 24 h and 72 h physiology/trajectories, in terms of discrimination and calibration, and to assess clinical utility using decision-curve analysis [22]. Model development and internal validation were conducted and reported in line with established guidance for prediction modeling studies [20,25].
3. Use explainability methods (e.g., SHAP value analysis and partial dependence plots) to identify which trajectories are most strongly associated with poor outcome and to derive intuitive, bedside-relevant risk profiles [18,19].
4. Discuss how such models might eventually be integrated into the ICU doctor’s daily workflow as a complement—rather than a replacement—for neurological examination and multidisciplinary discussion [2,4,5,6,20].

We hypothesize that progressively updated trajectory-based models using simple, routinely collected data may provide additional time-updated prognostic information compared with earlier-stage assessments while recognizing that observed performance differences require cautious interpretation because of the modest sample size, informative follow-up measurement patterns, and internal-validation-only design.

We additionally prespecified robustness analyses to assess two key methodological vulnerabilities in small ICU machine-learning studies, including severity-proxy sensitivity and label-permutation sanity checks. Specifically, robustness analyses targeted (i) dependence on organ-support intensity markers (feature-exclusion sensitivity analyses) and (ii) inadvertent evaluation leakage (label-permutation sanity checks), providing safeguards against overly optimistic performance estimates in modest single-centre cohorts [20,21,26,27].

## 2. Materials and Methods

### 2.1. Data Preparation and Database Construction

**Figure 1:**
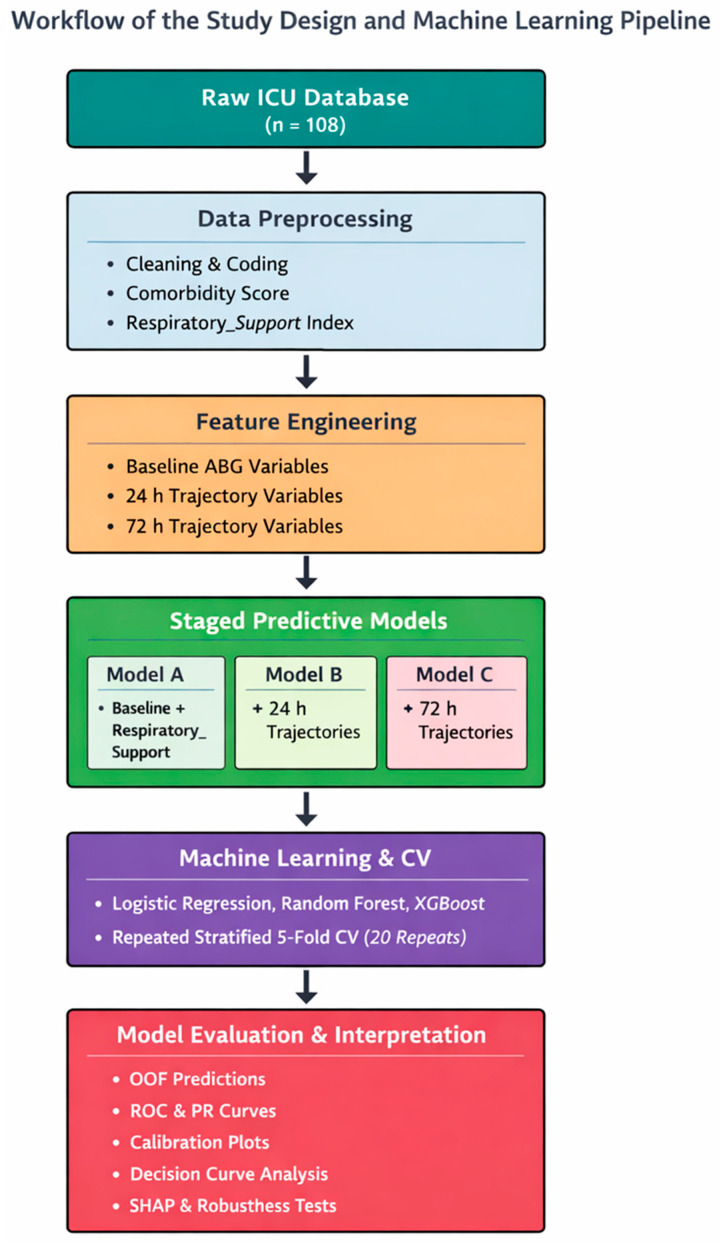
Workflow of the study design and machine-learning pipeline. The diagram illustrates the sequential steps of the analysis, including preprocessing of the ICU dataset, feature engineering of baseline and trajectory variables (24 h and 72 h), construction of staged predictive models (Model A–C), model training using logistic regression, random forest, and gradient boosting (XGBoost), repeated stratified cross-validation with aligned out-of-fold predictions, and evaluation using discrimination, calibration, decision-curve analysis, and explainability methods.

The initial dataset consisted of a raw clinical database collected from patients admitted to the intensive care unit (ICU). The original database contained demographic information, comorbidities, COVID-19 status, respiratory support variables, arterial blood gas measurements recorded at ICU admission, 24 h, and 72 h, as well as laboratory biomarkers and ICU outcome. The overall analytical workflow of the study, including data preprocessing, feature engineering, staged predictive modeling, cross-validation, and model evaluation, is summarized in Figure 1.

#### Study Population and Operational Definition of Coma

The study included adult ICU patients with clinically documented coma or unresponsiveness requiring intensive monitoring and supportive care. In the present study, “comatose” was used as an operational clinical descriptor based on the treating team’s documentation at ICU admission rather than a strictly etiology-specific neurological research classification. The cohort therefore reflects a heterogeneous real-world ICU population that may include structural, metabolic, infectious, hypoxic, or mixed causes of impaired consciousness. This pragmatic definition increases clinical realism but limits etiologic specificity and should be considered when interpreting model generalizability. Standardized admission Glasgow Coma Scale values, systematic etiologic categories of coma, sedation exposure, neuromuscular-blocker use, targeted temperature management (TTM), and explicit distinction between pharmacological unresponsiveness and neurological coma were not consistently available in the retrospective dataset. Therefore, the cohort should be interpreted as a pragmatic real-world ICU population with documented coma or unresponsiveness, rather than as an etiologically homogeneous neurocritical-care cohort. This limitation is particularly relevant because ABG/SpO_2_ trajectories may have different prognostic meanings in post-anoxic coma, stroke, traumatic brain injury, sepsis-associated encephalopathy, metabolic coma, intoxication, and mixed etiologies.

### 2.2. Data Cleaning and Quality Control

Data preprocessing was performed in Microsoft Excel prior to statistical analysis. The raw dataset was stored in Sheet1, while Sheet2 was used to generate the analytical dataset through formula-based transformation of the original values. This approach ensured that the processed dataset remained dynamically linked to the original data while preserving traceability of all transformations.

All variables were systematically inspected for data integrity, consistency of format, and missing values. Cells containing spreadsheet error values (e.g., #NULL!) in the raw dataset were interpreted as missing measurements and treated as missing data. These error values were converted to blank cells in the analytical dataset in order to prevent their propagation through subsequent calculations. No artificial zero imputation was performed for physiological or laboratory variables because zero values would represent physiologically implausible measurements.

Missing values were visually inspected using conditional formatting to highlight blank cells across the dataset. The number and distribution of missing observations were subsequently verified for each variable before analysis.

In addition, data types were verified to ensure that continuous variables were stored in numeric format and that categorical variables were properly encoded.

Prior to statistical modeling, an additional preprocessing step was performed in Python 3.12.13 (Google Colab) to standardize variable names and ensure compatibility with statistical and machine-learning libraries. All variable headers were converted to lowercase characters with underscore separators, and special characters (such as spaces or Unicode symbols) were removed. For example, “SpO_2_ at ICU admission” was converted to *spo2_0*, “Arterial pH at admission” to *ph_0*, and analogous naming conventions were applied to the 24 h and 72 h measurements. This standardization ensured reproducibility and facilitated automated data processing.

### 2.3. Variable Coding and Transformation

Categorical variables were converted into numerical form to enable statistical and machine-learning analyses. Binary variables were encoded using a 0/1 scheme, where 0 represented absence of the condition and 1 represented presence.

Sex was coded as 0 for female and 1 for male. Comorbidities including prior stroke, chronic kidney disease, obesity, chronic ischemic heart disease, chronic heart failure, atrial fibrillation, hypertension, and diabetes mellitus were each coded as binary variables (0 = no, 1 = yes).

To quantify the overall burden of chronic disease, a comorbidity_score variable was constructed as the sum of the individual comorbidity indicators. This composite score ranged from 0 to 8, representing the total number of documented comorbid conditions for each patient.

COVID-19 status was extracted from the original textual entries describing infection status at hospital admission. Text strings beginning with “Da” (yes) were coded as 1, indicating confirmed infection prior to admission, while entries beginning with “Nu” (no) were coded as 0.

The ICU outcome variable was derived from the discharge status recorded in the database. Patients labeled “Deces” were coded as 1 (ICU death), whereas patients labeled “Transfer secție” were coded as 0 (survival with transfer to a hospital ward). This endpoint reflects ICU-course outcome rather than long-term survival or neurological recovery.

Continuous variables, including age, arterial blood gas parameters (SpO_2_, arterial pH, PaCO_2_, PaO_2_, bicarbonate), and laboratory markers (leukocytes, neutrophils, C-reactive protein, procalcitonin, lactate dehydrogenase, D-dimer, international normalized ratio, and activated partial thromboplastin time), were retained in their original numeric format. Measurements were recorded at ICU admission and at subsequent time points (24 h and 72 h).

### 2.4. Handling of Respiratory Support Variables

Respiratory support modalities documented in the original dataset included nasal cannula oxygen therapy, face mask oxygen therapy, high-flow nasal cannula, non-invasive ventilation, and invasive mechanical ventilation. These variables were initially retained as binary indicators (0 = absent, 1 = present) in the analytical dataset.

To reduce dimensionality and better represent the clinical severity of respiratory support, a derived variable termed respiratory_support was created. This ordinal variable summarized the highest level of respiratory assistance required by each patient and was coded as follows:

- 0—conventional oxygen therapy (nasal cannula or face mask)
- 1—advanced non-invasive respiratory support (high-flow nasal cannula or non-invasive ventilation)
- 2—invasive mechanical ventilation

The respiratory_support index represented the highest level of respiratory support required during the first 72 h of ICU stay. Because this variable may function as an organ-support intensity proxy, we prespecified sensitivity analyses repeating all models after excluding respiratory_support (Section 2.9). Admission-specific mechanical ventilation status and total duration of invasive ventilation were not consistently available as separate variables; therefore, respiratory_support should be interpreted as a 72 h support-intensity summary rather than a baseline ventilation-status variable.

This transformation allowed the different oxygen delivery modalities to be represented by a single clinically interpretable severity scale while minimizing collinearity between multiple respiratory support variables.

### 2.5. Derived Physiological Trajectory Variables

Because arterial blood gas measurements were available at multiple time points, additional derived variables were created to capture early physiological evolution during the first 72 h of ICU stay. These trajectory variables represented the difference between follow-up and baseline measurements.

Specifically, change variables were calculated for arterial pH, PaO_2_, PaCO_2_, SpO_2_, and bicarbonate at both 24 h and 72 h relative to admission values. These variables captured short-term physiological trends that may reflect clinical deterioration or recovery.

ABG measurements were extracted according to the clinically recorded ICU admission, 24 h, and 72 h timepoints. Exact clock-time deviations from these nominal timepoints and whether individual samples were obtained strictly by protocol or triggered by clinical events were not consistently available in the retrospective dataset. Therefore, the derived trajectory variables should be interpreted as pragmatic clinical time-window summaries rather than uniformly timed protocol-based measurements.

During the final preprocessing stage, trajectory variables were verified to ensure internal consistency. Change variables were only retained when both the baseline and follow-up measurements were available. If either value was missing, the corresponding trajectory variable was recorded as missing. This validation step prevented incorrect difference calculations that could arise from spreadsheet formulas operating on incomplete observations.

### 2.6. Outlier Detection

Continuous variables were inspected for implausible values and potential data entry errors. Outlier detection was performed by reviewing value distributions and comparing them with physiologically plausible ranges reported in clinical practice.

Typical ranges considered during validation included:

- arterial pH approximately 7.0–7.7
- PaCO_2_ approximately 20–100 mmHg
- PaO_2_ approximately 30–300 mmHg
- SpO_2_ approximately 50–100%

Values outside these ranges were manually verified against the original dataset to confirm whether they represented true measurements or potential data entry errors.

### 2.7. Construction of the Analytical Dataset

The final analytical dataset consisted exclusively of cleaned numeric variables suitable for statistical modeling and machine learning analysis. The dataset included:

- demographic variables (age, sex),
- individual comorbidities and the derived comorbidity_score,
- COVID-19 status,
- arterial blood gas measurements at admission, 24 h, and 72 h,
- derived physiological trajectory variables,
- laboratory biomarkers at admission,
- respiratory support variables and the derived respiratory_support severity index, and
- ICU outcome.

All preprocessing steps were completed prior to statistical analysis to ensure consistency, reproducibility, and transparency of the dataset used for model development.

The final dataset was also inspected for patterns of missing observations after import into the statistical analysis environment. Follow-up arterial blood gas measurements were available for 82 of 108 patients (75.9%) at 24 h and for 66 of 108 patients (61.1%) at 72 h. These proportions reflect the clinical nature of ICU datasets, where follow-up measurements may be unavailable due to early discharge, transfer, or death. To account for this variability, two indicator variables (has_24 h_abg and has_72 h_abg) were created to explicitly capture the availability of follow-up physiological measurements during modeling.

Missing follow-up measurements were treated as missing (blank/NaN) and were not imputed as physiological zeros. For statistical comparisons, analyses used available-case data for each variable. For machine-learning models, missing continuous predictors were handled using median imputation performed within the training folds only (implemented as part of the scikit-learn pipeline) to avoid information leakage from the full dataset into model fitting. Median imputation was chosen as a conservative fold-wise strategy compatible with repeated cross-validation and heterogeneous missingness patterns. Last-observation-carried-forward (LOCF) imputation was not used because ABG measurements were available only at sparse nominal timepoints (ICU admission, 24 h, and 72 h), and carrying admission values forward could artificially suppress true physiological change. K-nearest-neighbour (KNN) imputation was not used because the cohort size was modest, missingness was outcome-associated, and distance-based imputation could introduce instability or information leakage if not tuned within a nested validation framework. Therefore, median imputation was used only within training folds, while trajectory variables were computed only when both baseline and follow-up measurements were available. In addition, trajectory variables (Δ features) were only computed/retained when both baseline and follow-up measurements were available; if either component was missing, the corresponding trajectory feature was set to missing to prevent invalid difference calculations.

Because follow-up ABG availability depended partly on clinical evolution, including early death, transfer to ward, ongoing ICU stay, and sampling intensity, missingness at 24 h and 72 h was considered potentially informative rather than missing completely at random. The availability indicators has_24 h_abg and has_72 h_abg were therefore created to preserve transparency regarding follow-up measurement patterns and to characterize the missing-data structure. However, in the staged machine-learning models implemented in the present notebook analysis, these indicators were not included in the primary staged predictor sets. Consequently, the 24 h and especially the 72 h trajectory models should be interpreted as time-updated models conditional on the clinical process that generated follow-up ABG measurements, rather than as purely biological trajectory models. Informative-missingness bias therefore remains possible and should be considered when interpreting later-stage model performance [21,26,27].

#### Availability of Established ICU Severity Scores

Established ICU severity scores, including SOFA, APACHE II, and SAPS II, were not available as recorded variables in the retrospective dataset. In addition, the component variables required to reconstruct these scores reliably were incomplete or unavailable, including FiO_2_ for PaO_2_/FiO_2_ estimation, platelet count, bilirubin, vasopressor or mean arterial pressure data, Glasgow Coma Scale, creatinine or urine output, electrolyte values, hematocrit, admission type, and selected chronic health-status variables. Therefore, direct benchmarking against SOFA, APACHE II, or SAPS II could not be performed in the present analysis. This limitation should be considered when interpreting the incremental value of the proposed trajectory-based models. A structured summary of unavailable variables and their consequences is provided in Supplementary Table S5.

### 2.8. Statistical Analysis and Predictive Modeling

#### 2.8.1. Descriptive Statistical Analysis

Descriptive statistics were performed to summarize the clinical and laboratory characteristics of the study population. Continuous variables were assessed for distribution and are reported as mean ± standard deviation for normally distributed variables or median with interquartile range (IQR) when normality assumptions were not met. Categorical variables are presented as absolute counts and percentages.

Group comparisons between patients who survived ICU admission and those who died during ICU stay were performed using appropriate statistical tests. Continuous variables were compared using either the Student’s *t*-test or the Mann–Whitney U test, depending on data distribution. Categorical variables were analyzed using the Chi-square test or Fisher’s exact test when expected cell counts were small. A two-sided *p*-value < 0.05 was considered statistically significant.

Effect sizes were calculated as Cohen’s d for continuous variables and as φ coefficient, Cramér’s V, or odds ratio for categorical comparisons depending on the contingency table structure.

#### 2.8.2. Predictive Modeling Strategy

To evaluate the prognostic value of progressively available clinical and physiological information, a staged predictive modeling strategy was implemented. Because respiratory_support was defined as the highest level of respiratory support required during the first 72 h, it was not treated as a pure admission-time predictor. Therefore, models excluding respiratory_support, hereafter referred to as NoRS models, were considered the main interpretive analyses for assessing the prognostic contribution of ABG/SpO_2_ and biomarker information.

Models including respiratory_support were retained as secondary severity-aware clinical-status models to quantify the influence of organ-support intensity on discrimination and clinical utility.

**Model A: Earlier-stage Model**

The first model incorporated demographic characteristics, comorbidities, the derived comorbidity_score, COVID-19 status, arterial blood gas parameters at admission, and baseline laboratory biomarkers. In the main interpretive version of this model, respiratory_support was excluded. A secondary severity-aware version additionally included respiratory_support, defined as the highest level of respiratory support during the first 72 h.

**Model B: Early Evolution Model (24 h)**

The second model incorporated all variables included in Model A and additionally integrated derived trajectory variables representing the change between admission and 24 h values. The main interpretive version excluded respiratory_support, while the secondary severity-aware version included respiratory_support. These included ΔSpO_2_ at 24 h, ΔpH at 24 h, ΔPaCO_2_ at 24 h, ΔPaO_2_ at 24 h, and Δbicarbonate at 24 h. This model captures early physiological evolution during the first day of intensive care treatment.

**Model C: Early Trajectory Model (72 h)**

The third model incorporated all variables from Model A and further integrated derived trajectory variables representing the change between admission and 72 h values. The main interpretive version excluded respiratory_support, while the secondary severity-aware version included respiratory_support. These included ΔSpO_2_ at 72 h, ΔpH at 72 h, ΔPaCO_2_ at 72 h, ΔPaO_2_ at 72 h, and Δbicarbonate at 72 h. This model reflects short-term physiological trajectories during the first 3 days of ICU care and allows evaluation of the additional prognostic information provided by early clinical evolution.

**Temporal availability constraint.** Each staged model was restricted to the variables defined for that stage. Models B and C are explicit time-updated prognostic models because they include 24 h and 72 h trajectory information, respectively. Any model including respiratory_support should also be interpreted as severity-aware because respiratory_support summarizes the highest organ-support level during the first 72 h. Therefore, respiratory_support-enriched models were not treated as strict admission-only prognostic models.

To address the possibility that model performance may be driven by simple admission variables rather than trajectory information, we additionally evaluated two parsimonious logistic-regression benchmark models using the same cross-validation framework: admission PaO_2_ alone, and a three-variable admission model that includes PaO_2_, LDH, and CRP. PaO_2_/FiO_2_ was not evaluated because FiO_2_ was not available and could not be reliably reconstructed from support-modality fields without recorded flow rates, ventilator settings, or inspired oxygen fraction.

To assess whether follow-up measurement availability itself contributed to the apparent time-updated signal, additional NoRS logistic-regression sensitivity models were fitted after adding has_24 h_abg and/or has_72 h_abg indicators to the corresponding staged models.

#### 2.8.3. Machine-Learning Models

Supervised machine-learning algorithms were applied to develop predictive models for ICU mortality. Multiple modeling approaches were considered in order to compare predictive performance across different methods. These included:

- Logistic regression, used as a baseline statistical model due to its interpretability and widespread use in clinical research [20,28].
- Random forest classifiers, which are ensemble-based tree models capable of capturing nonlinear relationships and interactions between variables [17].
- Gradient boosting classifiers, which iteratively improve predictive performance through sequential tree construction [18].

Model training was performed using the cleaned analytical dataset generated during preprocessing and implemented within a Python-based analysis pipeline (Google Colab). The pipeline included automated handling of missing values, feature scaling where applicable, and repeated stratified cross-validation to ensure robust estimation of model performance. To reduce the risk of overfitting given the modest dataset size, model performance was evaluated using cross-validation techniques. Gradient boosting was implemented using XGBoost classifiers with probabilistic outputs, and trained and evaluated under the same cross-validation and preprocessing pipeline as the other models. Given the modest sample size, logistic regression was treated as the primary interpretable reference model, while random forest and XGBoost were considered secondary nonlinear comparisons. Algorithm comparisons were interpreted descriptively rather than as definitive evidence of superiority of one model family over another.

##### Model Implementation Details

Models were evaluated using repeated stratified 5-fold cross-validation with 20 repeats (RepeatedStratifiedKFold), using a fixed random seed of 42. All preprocessing steps were performed within each training fold and then applied to the corresponding held-out fold to minimize information leakage. Continuous predictors were imputed using median imputation in all models, and feature scaling with StandardScaler was applied for logistic regression only.

Logistic regression was implemented with a maximum of 5000 iterations and class_weight = “balanced”. Solver, penalty type, and regularization strength were not explicitly overridden in the notebook and therefore remained at their library default settings.

Random forest models were implemented with 600 trees (n_estimators = 600), class_weight = “balanced_subsample”, min_samples_leaf = 2, and random_state = 42. Maximum tree depth and minimum samples required for node splitting were not explicitly overridden and therefore remained at their default values.

Gradient boosting was implemented using XGBoost with probabilistic output (objective = “binary:logistic”), n_estimators = 600, learning_rate = 0.03, max_depth = 3, subsample = 0.9, colsample_bytree = 0.9, reg_lambda = 1.0, min_child_weight = 1.0, gamma = 0.0, eval_metric = “logloss”, random_state = 42, and n_jobs = −1.

Hyperparameters were prespecified in the analysis notebook and were not selected by GridSearch, RandomSearch, Bayesian optimization, or nested hyperparameter optimization within the cross-validation folds [27,28]. We acknowledge that systematic hyperparameter optimization and feature elimination can improve model comparison in larger datasets and has been successfully applied in other biomedical machine-learning contexts [29]. However, in the present modest retrospective cohort, extensive non-nested optimization would increase the risk of overfitting and optimistic performance estimation. Therefore, hyperparameters were prespecified, logistic regression was treated as the primary interpretable reference model, and random forest/XGBoost were interpreted as secondary exploratory nonlinear comparisons rather than definitive evidence of algorithmic superiority. No automated feature-selection procedure was applied before modeling. Candidate predictors were selected a priori based on clinical availability and physiological relevance. Therefore, model performance estimates, algorithm comparisons, and feature-importance rankings should be interpreted as exploratory. Thus, the present analysis prioritizes transparent, prespecified modeling over optimized algorithmic comparison.

#### 2.8.4. Model Evaluation

Model performance was assessed using standard classification metrics. Discriminatory ability was evaluated using the area under the receiver operating characteristic curve (AUC-ROC). For the primary NoRS logistic-regression models and additional sensitivity analyses, 95% confidence intervals for AUC-ROC were estimated by non-parametric bootstrap resampling of aligned out-of-fold predicted probabilities at the patient level. Because of the modest cohort size and outcome-associated follow-up availability, staged AUC differences were interpreted descriptively rather than as definitive evidence of model superiority. Additional performance metrics included accuracy, sensitivity, specificity, precision, F1 score, and the Brier score. Receiver operating characteristic (ROC) curves were constructed for each model to compare predictive performance across Model A, Model B, and Model C. Comparisons between Model A, Model B, and Model C were used to evaluate whether incorporating early physiological trajectories improved time-updated mortality risk estimation at 24 h and 72 h.

To obtain unbiased per-patient predictions for downstream calibration, decision-curve analysis, and explainability, model performance was estimated using repeated stratified cross-validation with aligned out-of-fold (OOF) predictions. Specifically, each patient received predictions only from folds in which that patient was held out; when repeated cross-validation was used, OOF predictions were averaged across repeats to yield one probability estimate per patient.

Calibration was evaluated using the Brier score and calibration curves [28,30,31]. Operating-point metrics (accuracy, sensitivity, specificity, precision) were computed using the Youden-index threshold derived from cross-validated predictions.

Clinical utility was assessed using decision curve analysis (DCA) [22] computed from OOF predicted probabilities across a clinically relevant range of threshold probabilities. Net benefit of each model was compared with default strategies of treating all and treating none. Additional methodological details regarding the aligned out-of-fold prediction framework, decision-curve analysis, XGBoost implementation, explainability analyses, and robustness/leakage-control procedures are provided in Supplementary Methods S1–S5.

#### 2.8.5. Interpretability and Clinical Relevance

To ensure clinical interpretability of the predictive models, we performed model-specific explainability analyses. Global feature contributions were quantified using SHAP (Shapley additive explanations) values [19] computed on the OOF modeling dataset and visualized using SHAP summary and global importance plots. To characterize the direction and potential nonlinearity of key predictors, partial dependence plots (PDPs) [28] were generated for the highest-ranked variables (e.g., oxygenation and inflammatory markers) and for selected trajectory features. Explainability analyses were performed primarily for the gradient-boosting and random forest models and were used to translate model behavior into clinically interpretable patterns consistent with bedside reasoning.

#### 2.8.6. Complete-Window Sensitivity Analyses and Sample-Size Adequacy Assessment

Because follow-up ABG availability was outcome-associated, additional complete-window sensitivity analyses were performed to directly assess whether trajectory variables added discriminatory information among patients with recorded follow-up measurements. First, analyses were restricted to patients with available 24 h ABG measurements (has_24 h_abg = 1), and within this subgroup a baseline-only NoRS logistic-regression model was compared with a baseline-plus-24 h trajectory NoRS logistic-regression model. Second, analyses were restricted to patients with available 72 h ABG measurements (has_72 h_abg = 1), and within this subgroup, a baseline-only NoRS logistic-regression model was compared with a baseline-plus-72 h trajectory NoRS logistic-regression model. All models used the same repeated cross-validation, fold-wise preprocessing, and aligned out-of-fold prediction framework as the main analyses. Because these subgroup analyses involved smaller and outcome-imbalanced samples, they were interpreted as exploratory.

Sample-size adequacy for prediction-model development was assessed using a Riley-framework-informed approach for binary outcomes, based on the observed outcome prevalence, the approximate number of candidate predictor parameters in each staged NoRS model, and the observed C-statistics. The resulting adequacy estimates were compared with the available cohort size to evaluate overfitting risk, events-per-predictor limitations, and the need for shrinkage-aware interpretation.

### 2.9. Robustness and Leakage Control Analyses

Because exceptionally high discrimination can raise concerns about unintended information leakage or dominance of a single severity proxy, two prespecified robustness checks were performed [21,26,27]. First, all staged models were re-evaluated after excluding respiratory_support from the feature set to quantify how strongly performance depended on this organ-support intensity marker. Second, a label-permutation sanity check was performed by randomly shuffling ICU outcome labels and repeating model evaluation under identical cross-validation settings. Under label permutation, discrimination collapsed toward chance level (AUC ≈ 0.55), supporting the absence of evaluation leakage.

Results of the exclusion sensitivity analysis are reported as Supplementary Table S1, and the label-permutation ROC curve is shown in Supplementary Figure S1. Additional supplementary tables and figures are numbered sequentially according to their first mention in the main text.

These robustness checks were performed for the main modeling families (logistic regression, random forest, and gradient boosting/XGBoost) using identical preprocessing and cross-validation settings.

## 3. Results

### 3.1. Study Population

The final analytical dataset consisted of 108 patients admitted to the intensive care unit (ICU). Among them, 71/108 (65.7%) died during ICU stay, while 37/108 (34.3%) survived and were transferred to a hospital ward. Baseline demographic, clinical, and laboratory characteristics are summarized in Table 1. The median age of the cohort was 72 years (IQR 17), corresponding to a mean age of 68.9 ± 14.4 years, and 62/108 (57.4%) were male. The median comorbidity score was 2 (IQR 1), indicating a moderate burden of chronic disease. At ICU admission, patients presented with significant respiratory impairment, with median SpO_2_ 85% and median PaO_2_ 58.8 mmHg. Inflammatory markers were elevated (median CRP 96 mg/L; median procalcitonin 0.70 ng/mL; Table 1).

### 3.2. Availability of Follow-Up Physiological Measurements

Complete ABG follow-up data were available for 82/108 patients (75.9%) at 24 h and for 66/108 patients (61.1%) at 72 h. Missing follow-up measurements reflected real-world ICU trajectories, including early death, transfer to ward, ongoing ICU stay without repeat sampling, or incomplete documentation. Complete 72 h ABG availability differed by outcome, being present in 43.2% of survivors and 70.4% of deaths, indicating outcome-associated follow-up measurement patterns.

This finding is important for interpretation of the time-updated models. Absence of a 72 h ABG should not be interpreted as random missingness. It may reflect clinical improvement followed by transfer to the ward, early death before repeat measurement, reduced sampling intensity, or other care-process factors. Therefore, later-stage models, particularly Model C, may partly reflect both physiological evolution and the clinical process determining who remained in ICU and under observation long enough to contribute 72 h measurements.

### 3.3. Comparison Between Survivors and Non-Survivors

Clinical and laboratory differences between ICU survivors and non-survivors are presented in Table 2. Patients who died during ICU stay exhibited more severe respiratory dysfunction compared with survivors. At ICU admission, non-survivors had lower PaO_2_ values than survivors (median 54 mmHg vs. 69.2 mmHg). Differences in oxygenation parameters became more pronounced during follow-up measurements. At 24 h, non-survivors showed lower PaO_2_ values (median 56.3 mmHg vs. 87 mmHg in survivors) and lower SpO_2_ values (median 92% vs. 95%). At 72 h, these differences persisted, with non-survivors continuing to exhibit poorer oxygenation status.

Laboratory biomarkers also differed between the two groups. Non-survivors had higher lactate dehydrogenase (LDH) and C-reactive protein (CRP) values than survivors. Coagulation abnormalities were also more pronounced in patients who died, including higher D-dimer and activated partial thromboplastin time (aPTT) values.

Respiratory support severity showed extreme separation by outcome (Table 2): respiratory_support = 2 occurred in 70/71 (98.6%) ICU deaths compared with 4/37 (10.8%) survivors, while respiratory_support = 0 occurred in 22/37 (59.5%) survivors and 0/71 (0%) deaths. This marked separation indicates that organ-support intensity was a dominant severity signal in the cohort and motivated the prespecified NoRS sensitivity models (Section 3.7).

### 3.4. Univariate Associations with ICU Mortality

Univariate analyses are provided in Supplementary Table S4 for descriptive purposes only. The strongest univariate association was observed for respiratory_support, consistent with its near-complete separation of outcome groups. Oxygenation variables, particularly PaO_2_ at admission, 24 h, and 72 h, as well as pH-related measures and SpO_2_ during follow-up, were also associated with ICU mortality after false-discovery-rate correction. Several laboratory biomarkers, including LDH, CRP, D-dimer, aPTT, and leukocyte count, also showed significant univariate associations. Because the objective of the study was prediction modeling rather than univariate risk-factor inference, these analyses were not used for automated feature selection and should be interpreted descriptively.

### 3.5. Predictive Model Performance

Three staged predictive models were developed to evaluate the prognostic information provided by progressively available clinical and physiological data during the first 72 h of ICU admission. Because respiratory_support was defined as the maximum respiratory-support level during the first 72 h and strongly separated outcome groups (Table 2), models excluding respiratory_support were treated as the primary interpretive analyses. Logistic regression was used as the primary model in the main text because of its interpretability, parsimony, and suitability for a modest cohort with a low effective events-per-variable ratio. Random forest and XGBoost results are retained as secondary exploratory methods-comparison analyses in Supplementary Tables S1–S3.

Primary NoRS logistic-regression performance is summarized in Table 3. Model A_noRS, based on admission variables without respiratory_support, achieved AUC-ROC 0.822 (95% CI 0.739–0.897), PR-AUC 0.889, and Brier score 0.167. Model B_noRS, incorporating 24 h trajectory variables, achieved AUC-ROC 0.848 (95% CI 0.766–0.919), PR-AUC 0.909, and Brier score 0.155. Model C_noRS, incorporating 72 h trajectory variables, achieved AUC-ROC 0.895 (95% CI 0.830–0.951), PR-AUC 0.942, and Brier score 0.126. These results suggest increasing time-updated prognostic information across staged models, but the overlapping confidence intervals and modest cohort size require cautious interpretation.

Respiratory_support-enriched models are reported in Supplementary Table S6 and retained only as secondary severity-aware analyses. Because respiratory_support was defined as the highest respiratory-support level during the first 72 h and strongly separated outcome groups, these models should not be interpreted as strict admission-only prognostic models.

Simple benchmark models were added to contextualize the staged analyses. Admission PaO_2_ alone achieved AUC-ROC 0.788 (95% CI 0.698–0.871), PR-AUC 0.865, and Brier score 0.185. A parsimonious three-variable admission model using PaO_2_, LDH, and CRP achieved AUC-ROC 0.862 (95% CI 0.787–0.929), PR-AUC 0.920, and Brier score 0.150. The primary Model C_noRS logistic-regression model remained numerically higher, with AUC-ROC 0.895 (95% CI 0.830–0.951), PR-AUC 0.942, and Brier score 0.126. These comparisons are exploratory and should not be interpreted as definitive superiority of the trajectory-enriched model. Detailed simple benchmark-model performance is provided in Supplementary Table S8.

#### 3.5.1. Complete-Window Trajectory Sensitivity Analyses

To directly test whether trajectory variables added discriminatory information among patients with recorded follow-up ABG measurements, complete-window sensitivity analyses were performed. In the 24 h ABG-available subgroup (has_24 h_abg = 1; n = 82; ICU deaths = 57), the baseline-only NoRS logistic-regression model achieved AUC-ROC 0.857 (95% CI 0.751–0.937), while the baseline-plus-24 h trajectory NoRS model achieved AUC-ROC 0.879 (95% CI 0.776–0.956). Thus, the addition of 24 h trajectory features produced only a small numerical increase in discrimination, with substantially overlapping confidence intervals.

In the 72 h ABG-available subgroup (has_72 h_abg = 1; n = 66; ICU deaths = 50), the baseline-only NoRS logistic-regression model achieved AUC-ROC 0.759 (95% CI 0.602–0.888), while the baseline-plus-72 h trajectory NoRS model achieved AUC-ROC 0.940 (95% CI 0.876–0.984). This analysis showed a larger numerical increase after addition of 72 h trajectory features. However, because the subgroup was smaller and outcome-imbalanced, this result should be interpreted as exploratory and hypothesis-generating rather than definitive evidence of incremental trajectory value. Detailed complete-window sensitivity results are provided in Supplementary Table S9.

#### 3.5.2. Sample-Size Adequacy Assessment

A Riley framework-informed sample-size adequacy assessment indicated that the available cohort did not meet conservative requirements for stable development of the staged NoRS prediction models. Model A_noRS included 16 candidate predictors, with 71 events and 37 non-events, corresponding to 4.44 events per predictor and 2.31 non-events per predictor. The approximate required total sample size was 935 patients. Models B_noRS and C_noRS each included 21 candidate predictors, corresponding to 3.38 events per predictor and 1.76 non-events per predictor, with an approximate required total sample size of 1226 patients. Therefore, the available cohort of 108 patients did not meet the calculated adequacy requirements for definitive prediction-model development. These findings support interpreting all staged comparisons, calibration patterns, and feature-importance rankings as exploratory and potentially affected by overfitting and limited shrinkage. The sample-size adequacy assessment is provided in Supplementary Table S10.

### 3.6. Discrimination, Calibration, and Precision–Recall Performance

**Figure 2:**
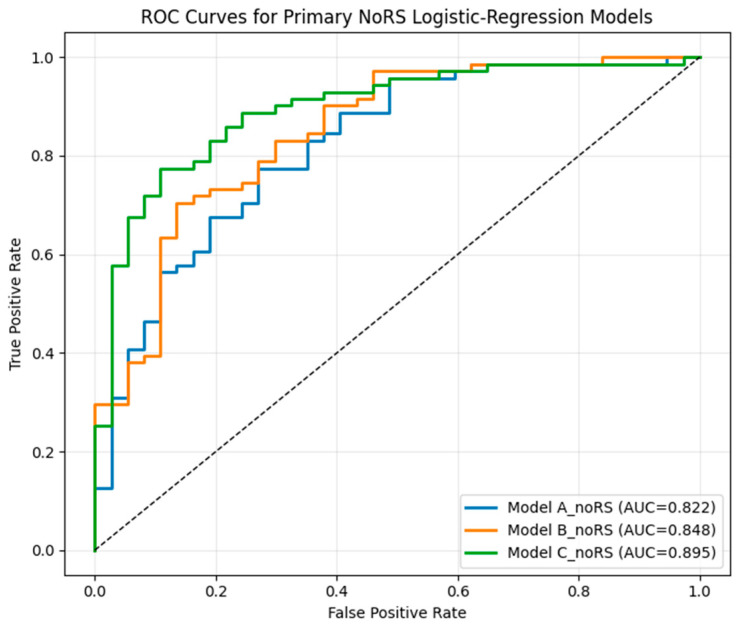
Receiver operating characteristic (ROC) curves for the primary NoRS logistic-regression models based on aligned out-of-fold predictions. These models exclude respiratory_support and represent the main interpretive analyses. Model A_noRS includes admission variables without the 72 h respiratory-support summary. Model B_noRS, additionally, includes 24 h trajectory features, and Model C_noRS includes 72 h trajectory features. The corresponding AUC-ROC values were 0.822, 0.848, and 0.895, respectively.

**Figure 3:**
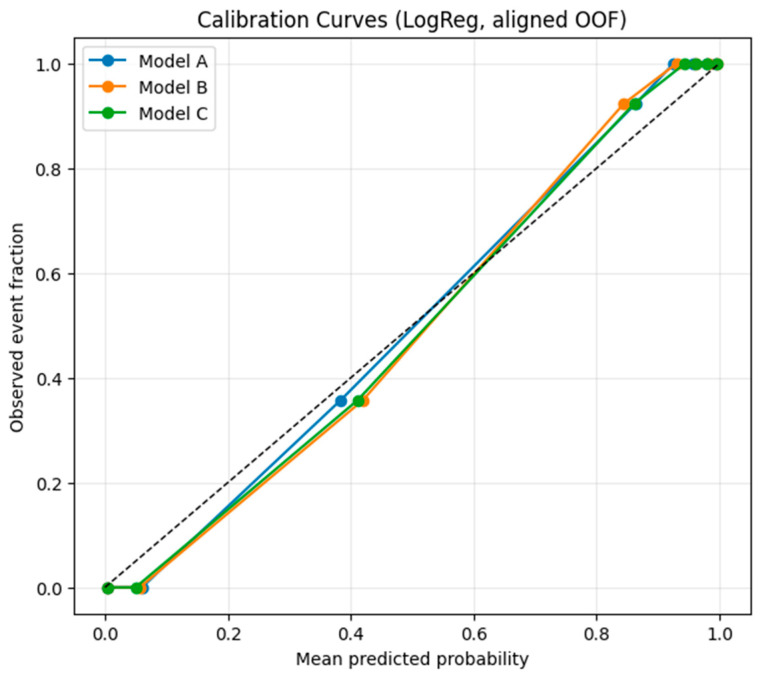
Calibration curves for logistic regression models predicting ICU mortality based on aligned out-of-fold predictions. The diagonal dashed line represents perfect calibration.

**Figure 4:**
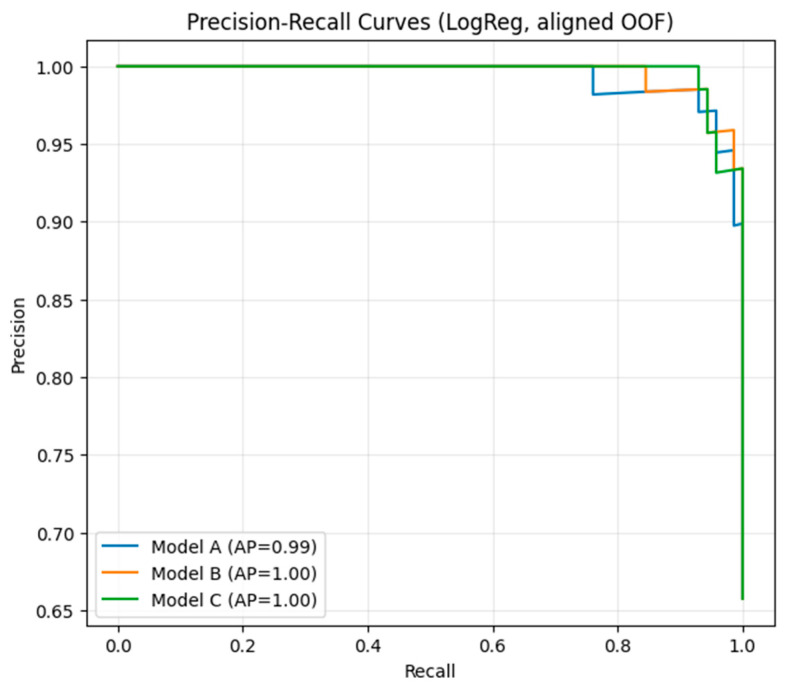
Precision–recall curves for logistic regression models predicting ICU mortality based on aligned out-of-fold predictions. High average precision indicates strong performance in identifying ICU mortality.

Receiver operating characteristic (ROC) curves for the primary NoRS logistic-regression models are shown in Figure 2. These curves correspond to the main interpretive analyses reported in Table 3 and exclude respiratory_support. Discrimination increased numerically from Model A_noRS to Model C_noRS, but these staged differences should be interpreted cautiously because of the modest sample size, overlapping confidence intervals, and outcome-associated follow-up measurement patterns. Calibration curves for logistic regression models are shown in Figure 3, demonstrating good agreement between predicted and observed outcome probabilities across risk strata. A complementary binned calibration plot illustrating the relationship between predicted probability and observed event frequency across prediction bins is provided in Supplementary Figure S16. Precision–recall curves are shown in Figure 4, demonstrating high average precision consistent with strong performance in identifying ICU deaths despite class imbalance.

#### Operating-Point Confusion Matrices for Model C

To complement discrimination, calibration, and decision-curve analyses, operating-point confusion matrices were added for Model C using the Youden-index threshold derived from aligned out-of-fold predictions. These matrices provide the absolute numbers of true positives, false negatives, true negatives, and false positives, allowing direct inspection of prediction errors.

For the respiratory_support-enriched Model C, both logistic regression and random forest correctly classified 66/71 ICU deaths and 37/37 ward-transfer survivors at the selected operating point (Table 4). However, these results should be interpreted cautiously because respiratory_support strongly separated outcome groups. In the clinically more informative NoRS Model C, logistic regression correctly classified 55/71 ICU deaths and 33/37 ward-transfer survivors, while random forest correctly classified 63/71 ICU deaths and 29/37 ward-transfer survivors (Table 4). These NoRS confusion matrices provide a more transparent view of the residual prediction errors after removal of the dominant organ-support severity proxy.

### 3.7. Clinical Utility and Robustness Analyses

#### 3.7.1. Decision-Curve Analysis

**Figure 5:**
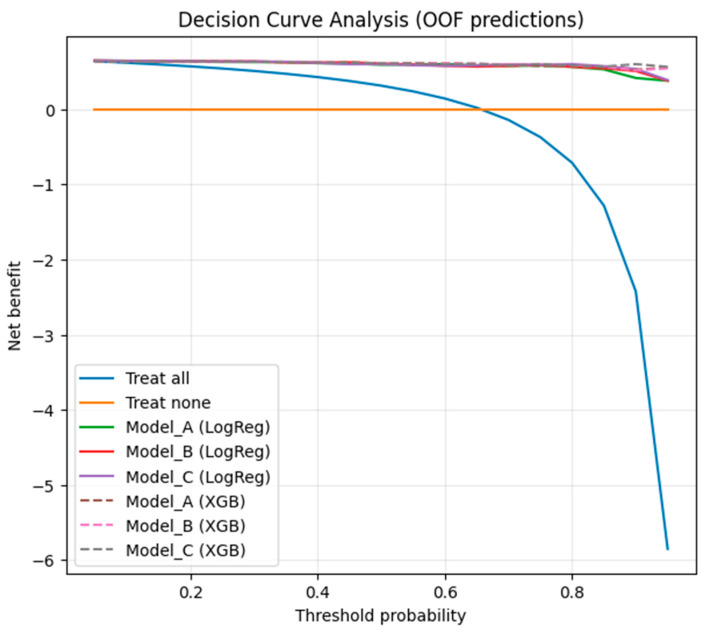
Decision curve analysis (DCA) using aligned out-of-fold predictions. Net benefit of each staged model is compared with “treat all” and “treat none” strategies across threshold probabilities. Curves are shown for logistic regression and XGBoost implementations of Models A–C.

Clinical utility was assessed using decision curve analysis (DCA) computed from aligned OOF probabilities. Across a clinically plausible range of threshold probabilities, staged models yielded higher net benefit than default strategies of treating all and treating none (Figure 5). Net benefit was numerically greatest for trajectory-enriched models, suggesting potential clinical utility when early evolution is available, but these findings remain exploratory and should not be interpreted as definitive evidence of incremental clinical value.

XGBoost and logistic regression produced largely overlapping net benefit profiles, consistent with their similar discrimination and calibration. Clinically, lower threshold probabilities may correspond to situations in which clinicians prioritize sensitivity and use the model output to trigger closer monitoring, repeat multidisciplinary review, or more frequent reassessment. Higher threshold probabilities may correspond to more consequential decisions, such as escalation discussions, treatment-limitation considerations, or transition toward palliative-care pathways. Because these thresholds are ethically, institutionally, and patient-context dependent, the DCA results should be interpreted as demonstrating potential net benefit across a plausible range of clinical thresholds rather than prescribing a universal decision cutoff. The corresponding NoRS XGBoost decision-curve analysis is provided in Supplementary Figure S15.

#### 3.7.2. Sensitivity Analyses Related to Respiratory_Support and Follow-Up ABG Availability

Because respiratory_support showed extreme separation by outcome (Table 2), models excluding respiratory_support were treated as the primary interpretive analyses and are reported in Table 3. These NoRS models were used to assess whether ABG/SpO_2_ and biomarker information retained prognostic information after removal of the dominant organ-support severity proxy.

In the primary NoRS logistic-regression models, Model A_noRS achieved AUC-ROC 0.822, Model B_noRS achieved AUC-ROC 0.848, and Model C_noRS achieved AUC-ROC 0.895. Random forest and XGBoost NoRS results are reported as secondary exploratory methods-comparison analyses in Supplementary Tables S1 and S3. These supplementary analyses showed broadly similar moderate-to-strong discrimination after removal of respiratory_support, supporting the robustness of the NoRS findings while avoiding claims of algorithmic superiority.

Because follow-up ABG availability was outcome-associated, additional NoRS logistic-regression sensitivity analyses included measurement-availability indicators. Model B_noRS with has_24 h_abg showed similar discrimination to the original Model B_noRS (AUC-ROC 0.845, 95% CI 0.765–0.916, versus 0.848 without the indicator). Model C_noRS with has_72 h_abg showed slightly higher discrimination than the original Model C_noRS (AUC-ROC 0.907, 95% CI 0.847–0.961, versus 0.895 without the indicator). Model C_noRS, including both has_24 h_abg and has_72 h_abg, achieved AUC-ROC 0.901 (95% CI 0.836–0.958). These results indicate that measurement availability did not abolish the time-updated signal, but later-stage performance may partly reflect care-process structure in addition to physiological evolution. Detailed measurement-availability sensitivity results are provided in Supplementary Table S7.

#### 3.7.3. Label-Permutation Sanity Check (Leakage Detection)

To evaluate whether the modeling pipeline could yield spuriously inflated performance due to leakage or evaluation artifacts, a label-permutation test was performed by randomly shuffling ICU outcome labels and repeating model evaluation under identical cross-validation settings. Under label permutation, discrimination collapsed toward chance (AUC-ROC 0.548 for logistic regression in Model A; Supplementary Figure S1), supporting the absence of systematic leakage and confirming that observed performance reflects genuine predictor signal.

### 3.8. Explainability: SHAP and Partial Dependence Analyses

To translate model behaviour into clinically interpretable patterns, we performed SHAP and partial dependence analyses for gradient boosting models.

In respiratory_support-enriched Model C (XGBoost), SHAP analyses showed that respiratory_support dominated global feature contributions, consistent with its strong separation by outcome (Table 2). Among non-support variables, baseline oxygenation markers, particularly SpO_2_ and PaO_2_, inflammatory burden represented by LDH, and oxygenation trajectory represented by ΔPaO_2_ at 72 h were prominent contributors (Supplementary Figures S2–S6). Partial dependence plots showed clinically coherent patterns: higher respiratory_support increased predicted mortality risk, lower admission SpO_2_ increased risk, and higher LDH increased risk with a plateau at high values (Supplementary Figures S7–S9).

In NoRS Model C (XGBoost), feature attribution shifted toward physiological and biomarker drivers. Admission PaO_2_, LDH, ΔPaO_2_ at 72 h, admission pH, and admission SpO_2_ emerged as the dominant contributors (Supplementary Figures S10 and S11). Partial dependence plots remained clinically coherent: lower PaO_2_ at admission, higher LDH, and unfavorable oxygenation trajectories, particularly failure of ΔPaO_2_ at 72 h to improve, were associated with higher predicted mortality (Supplementary Figures S12–S14).

## 4. Discussion

### 4.1. Principal Findings

In this retrospective single-centre cohort of 108 ICU patients with clinically documented coma or unresponsiveness, we developed staged, time-updated prognostic models using routine baseline physiology (ABG/SpO_2_), admission biomarkers, and derived physiological trajectory features at 24 h and 72 h. Across staged models, discrimination and calibration were clinically informative, and model behaviour remained interpretable through explainability analyses. The strongest empirical signal in the dataset was organ-support intensity, operationalized by the ordinal respiratory_support variable. This feature produced extreme separation between outcome groups, as shown in Table 2, where respiratory_support = 2 occurred in 70/71 ICU deaths and 4/37 survivors. This near-complete separation explains the exceptionally high AUC values in the respiratory_support-enriched models. However, because respiratory_support summarized the highest level of respiratory support during the first 72 h, these models were interpreted only as secondary severity-aware clinical-status analyses and not as admission-only prediction models. Importantly, when respiratory_support was excluded, the primary NoRS logistic-regression models retained moderate-to-strong discrimination, and trajectory-enriched models showed numerically higher performance than earlier-stage models. These findings suggest a possible time-updated physiological signal, particularly at 72 h, but complete-window sensitivity analyses and comparison with parsimonious admission-only benchmark models indicate that definitive incremental prognostic value from trajectory variables was not established in this cohort. Therefore, the staged performance pattern should be interpreted as exploratory and hypothesis-generating rather than as proof that trajectory variables independently improve prognostic performance.

Clinically, the models converged on a coherent set of bedside-relevant drivers: (i) oxygenation status at admission and over time (PaO_2_/SpO_2_ and ΔPaO_2_), (ii) inflammatory burden (LDH and CRP), and (iii) acid–base balance (pH), which aligns with how intensivists assess deterioration versus recovery during the first ICU days. The SHAP/PDP analyses further translated these associations into directional, clinically intuitive patterns: poorer oxygenation and rising inflammatory markers increased predicted mortality risk, while favourable oxygenation trajectories reduced risk. These findings reinforce the conceptual motivation of the study: trajectory-based risk estimation is aligned with ICU reasoning because it reflects not only baseline severity but also early response (or non-response) to treatment.

Accordingly, the principal comparison in this study is best understood not as baseline-only versus longitudinal prediction in the strict temporal sense, but as progressively updated prognostic assessment over the first 72 h of ICU care.

The similar performance of logistic regression and nonlinear ensemble models suggests that, in this modest cohort, the main signal was driven by clinically interpretable severity and trajectory variables rather than by complex algorithmic interactions.

### 4.2. Interpretation of High Discrimination and Leakage Concerns

Exceptionally high discrimination in small clinical ML cohorts commonly triggers concerns about unintended leakage, overfitting, or dominance of an implicit outcome proxy [21,26,27,28]. In this dataset, respiratory_support—defined as the maximum support level during the first 72 h—functioned as a strong organ-support intensity marker and nearly stratified the cohort into a low-support group with predominantly survival and a high-support group with predominantly death. Specifically, level 2 respiratory support was present in 70/71 ICU deaths and only 4/37 survivors (Table 2). Clinically, this likely reflects a real severity gradient captured in a compact variable, although the potential influence of treatment-escalation decisions, non-escalation decisions, and the evolving dying trajectory cannot be excluded. Methodologically, because respiratory_support aggregates treatment intensity over a time window, models including this variable should be interpreted only as secondary severity-aware clinical-status models rather than as admission-time prognostic models.

The NoRS analyses are therefore central to the interpretation of the study. By excluding respiratory_support, these models evaluate whether routine ABG/SpO_2_ variables, biomarkers, and early trajectories retain prognostic information after removal of the dominant organ-support severity proxy. In the primary NoRS logistic-regression models, discrimination remained moderate-to-strong, and the 72 h trajectory model showed numerically higher discrimination than earlier-stage models. This supports a narrower and more defensible conclusion: early oxygenation and acid–base trajectories may contain time-updated prognostic information beyond the respiratory_support variable, but this signal should be interpreted cautiously because of the modest sample size and outcome-associated follow-up measurement patterns.

To address methodological concerns, we implemented robustness procedures commonly used as sanity checks in clinical ML studies [21,26,27,28]. First, exclusion of respiratory_support quantified the dependence of model performance on the dominant organ-support proxy and motivated the NoRS models as the primary interpretive analyses. Second, label-permutation testing was used to assess whether the modeling pipeline could produce spuriously inflated discrimination under randomized outcome labels. Under label permutation, discrimination collapsed toward chance (AUC ≈ 0.55), supporting the absence of systematic evaluation leakage. However, this sanity check does not eliminate the separate concern that time-window variables may encode care-process information; for this reason, respiratory_support-enriched models were demoted to secondary analyses, and follow-up measurement-availability sensitivity analyses were added.

Decision-curve analysis further showed that, at least internally, risk scores derived from aligned OOF predictions yielded positive net benefit across clinically plausible threshold probabilities, rather than producing high discrimination without clinical utility. Decision-curve analysis is a standard framework for evaluating whether a prediction model improves decision-making compared with treating all or treating none [22]. These findings support the potential clinical relevance of the internally validated risk estimates, but they do not establish clinical deployment readiness without external validation.

### 4.3. Interpretation of Time-Updated Physiological Trajectories

A key contribution of this work is the structured evaluation of risk estimates as early physiology evolves. In the primary NoRS analyses, trajectory-enriched models showed numerically higher discrimination than earlier-stage models, particularly at 72 h. However, the additional complete-window sensitivity analyses provide a more cautious interpretation of this pattern. Among patients with recorded 24 h ABG measurements, adding 24 h trajectory features produced only a small numerical increase in discrimination compared with the baseline-only NoRS model, with substantially overlapping confidence intervals. Among patients with recorded 72 h ABG measurements, adding 72 h trajectory features produced a larger numerical increase in discrimination; however, this subgroup was smaller and outcome-imbalanced, and the result should therefore be interpreted as exploratory rather than definitive evidence of independent incremental value.

Therefore, the staged approach should be understood as a sequence of time-updated reassessment points rather than as a single admission-time classifier. Within this framework, trajectory variables may serve as compact summaries of early physiological response or non-response to treatment. Nevertheless, the present data do not definitively demonstrate that trajectory-enriched models provide incremental prognostic value beyond simpler admission-only benchmarks. In particular, the parsimonious three-variable admission model using PaO_2_, LDH, and CRP achieved AUC-ROC 0.862, while Model C_noRS achieved AUC-ROC 0.895, with substantially overlapping confidence intervals. This overlap indicates that the added value of the full trajectory-enriched model over a simpler admission-only model was not established in this cohort.

Accordingly, the trajectory findings should be interpreted as hypothesis-generating evidence of a possible time-updated physiological signal, especially at 72 h, rather than as proof that trajectory variables independently improve prognostic performance. The 72 h model should also be interpreted in light of outcome-associated ABG availability, because the presence or absence of follow-up measurements may itself encode clinical trajectory and care-process information. Larger prospective cohorts with prespecified landmark analyses among patients alive and still in ICU at each time window are required to determine whether ABG/SpO_2_ trajectories provide reproducible incremental value beyond parsimonious baseline models.

### 4.4. Explainability: Translating Predictors into Bedside Phenotypes

Explainability was not treated as an “add-on,” but as a mechanism for mapping model predictions into physiologically interpretable statements. In the primary XGBoost models, SHAP summaries indicated [19] that respiratory_support dominated global importance, consistent with Table 2. When that feature was removed (NoRS), SHAP rankings shifted toward physiologic and biomarker predictors—particularly admission PaO_2_, LDH, ΔPaO_2_ at 72 h, admission pH, and admission SpO_2_—demonstrating that the model still discovers coherent medical structure when the strongest severity marker is excluded. Partial dependence plots were also clinically consistent: worsening oxygenation and increasing LDH were associated with increased predicted mortality risk, with evidence of non-linear effects and plateauing at extreme values in some predictors.

Beyond interpretability, these plots provide an explicit clinical plausibility check by addressing whether the model behaves in a manner consistent with expected ICU reasoning, which is an important criterion for acceptability of ML in high-stakes prognostication.

### 4.5. Comparison with Prior Work

Two bodies of the literature are most relevant: (i) traditional ICU severity scoring and (ii) modern ICU machine-learning prognostication using EHR data. Classic severity scores (e.g., APACHE/SAPS/SOFA) [7,8,9,10] were designed for broad ICU risk stratification and are often built from static or worst-value windows rather than explicit short-horizon physiological trajectories, which limits their bedside interpretability for “day-to-day change” in comatose patients. Modern ICU ML, particularly in large open datasets (e.g., MIMIC) [11,12,13,14,15,16], demonstrates that time-resolved physiological information improves mortality prediction and enables more flexible modelling—but often at the cost of high-dimensional features, missingness complexity, and limited transparency. Large ICU time-series benchmarking studies have further emphasized the importance of explicit temporal task definition, irregular sampling, missing-data handling, and standardized validation frameworks [32]. Methodological reviews of clinical prediction models have emphasized that strong apparent internal performance may not translate across settings, and that calibration, external validation, transparent reporting, and clinical-utility assessment are essential before model deployment [33].

The present study sits between these paradigms: it uses low-cost routinely collected ABG/SpO_2_ trajectories and a small biomarker panel while incorporating explainability (SHAP/PDP) and clinical utility analysis (DCA) [22], which are increasingly expected components for clinically oriented ML modelling. Table 5 summarizes the position of the present study relative to traditional ICU severity scores, large-scale EHR-based ICU ML, and neurocritical-care ML approaches.

The contribution of the present study should therefore be interpreted as incremental and pragmatic rather than as a fundamentally new machine-learning methodology: its value lies in applying interpretable, time-updated modeling to routinely available ABG/SpO_2_ trajectories in a clinically recognizable comatose ICU population. We therefore do not claim methodological novelty in time-series modeling; rather, the contribution is the pragmatic evaluation of sparse, routinely available ABG/SpO_2_ trajectory features in a clinically recognizable comatose ICU cohort.

### 4.6. Clinical Implications

If externally validated, this framework could support ICU teams in three practical ways:

(1) Risk updates at 24 h and 72 h that quantify whether early physiology is trending toward recovery or deterioration;
(2) Explainable individual predictions to support multidisciplinary discussion and family communication (why risk is high/low);
(3) Threshold-aware decision support (DCA) that links predicted probabilities to net benefit across realistic risk thresholds, rather than relying solely on AUC.

Any use of threshold-based outputs should remain subordinate to multidisciplinary clinical judgement, neurological examination, patient/family goals, and institutional ethics procedures.

Because prognostication in coma carries ethical risk of self-fulfilling prophecy, the intended use should be stated explicitly: the model is a decision-support adjunct that provides transparent summaries of physiology and early response, not a replacement for neurological examination or multimodal prognostication pathways recommended in post-cardiac-arrest care [2,4,5,6].

### 4.7. Limitations

This study has several limitations:

1. Single-centre retrospective design with modest sample size limits generalizability [21,26,27,28]. Established ICU severity scores such as SOFA, APACHE II, and SAPS II were not available as recorded variables and could not be reliably reconstructed from the retrospective dataset. Therefore, the present models were not benchmarked against validated ICU risk scores, and their incremental value over established severity scoring remains unproven. Future prospective validation should include direct comparison with SOFA, APACHE II, and/or SAPS II.
2. The sample size was modest relative to the number of candidate predictors, particularly in trajectory-enriched models. This limitation is consistent with methodological guidance emphasizing that clinical prediction-model development requires adequate sample size not only for discrimination but also for calibration stability and avoidance of overfitting [34]. Although repeated cross-validation and aligned out-of-fold predictions reduce optimism in internal performance estimation, they do not eliminate instability caused by limited effective sample size [27,28]. No automated feature-selection procedure, LASSO penalization, principal-component analysis (PCA), or recursive feature elimination (RFE) was applied before modeling, and the effective events-per-variable ratio may therefore be low in trajectory-enriched models. Candidate predictors were selected a priori based on clinical availability and physiological relevance, but model estimates, feature-importance rankings, calibration curves, and apparent differences between algorithms should be interpreted cautiously. Future studies should evaluate prespecified reduced predictor sets, LASSO or other penalized regression approaches, PCA/RFE-based feature reduction, or embedded feature-selection approaches within nested validation and external validation frameworks. In the present revision, bootstrap AUC confidence intervals were added to the primary NoRS logistic-regression models; therefore, staged AUC differences are interpreted descriptively rather than as definitive evidence of model superiority. The Riley framework-informed sample-size adequacy assessment further indicated that the cohort did not meet conservative requirements for stable prediction-model development, particularly for trajectory-enriched models.
3. The parsimonious three-variable admission model using PaO_2_, LDH, and CRP achieved AUC-ROC 0.862 (95% CI 0.787–0.929), whereas Model C_noRS achieved AUC-ROC 0.895 (95% CI 0.830–0.951). Because these confidence intervals overlapped substantially, the present study does not demonstrate definitive incremental prognostic value of the full trajectory-enriched model over a simpler admission-only benchmark. Therefore, the added modeling framework should be interpreted as exploratory and hypothesis-generating rather than as evidence that increased model complexity is clinically justified in the present cohort.
4. Outcome-associated missingness at 24 h and 72 h is a major limitation. Follow-up ABGs were not missing completely at random: availability depended partly on clinical trajectory, including early death, transfer to ward, ongoing ICU stay, and sampling intensity. Although measurement-availability indicators were created to characterize this structure, they were not included in the final fitted staged models. Additional sensitivity analyses, including has_24 h_abg and has_72 h_abg, were therefore performed to evaluate whether follow-up measurement availability contributed to model discrimination. These analyses showed that inclusion of measurement-availability indicators produced only limited changes in discrimination, although the slight increase observed in the 72 h model suggests that later-stage performance may still partly reflect care-process structure. Additional complete-window sensitivity analyses restricted to patients with recorded follow-up ABG measurements showed only a small numerical gain after adding 24 h trajectory features, whereas the 72 h complete-window analysis showed a larger numerical gain but in a smaller and outcome-imbalanced subgroup. Therefore, these results support only a hypothesis-generating trajectory-associated signal rather than definitive evidence of independent incremental value.
5. Therefore, part of the predictive signal in later-stage models may reflect care-process structure in addition to physiological evolution itself [21,26,27]. In particular, the 72 h model should be interpreted as a time-updated model conditional on follow-up data availability, not as a purely biological trajectory model. A formal landmark analysis would be preferable but could not be performed reliably because exact ICU-stay status, death timing, and ward-transfer timing relative to the nominal 24 h and 72 h ABG windows were not consistently available. Future studies should address this issue using prespecified landmark analyses among patients alive and still in ICU at 24 h and 72 h, explicit missingness-indicator sensitivity models, inverse-probability weighting, or joint modeling approaches for longitudinal physiology and survival. This limitation corresponds directly to the missing-data handling described in Section 2.7, where the measurement-availability indicators were created for transparency but were not included in the final fitted predictor sets.
6. Exact clock-time deviations for ABG sampling and whether samples were protocol-driven or event-driven were not consistently available. Therefore, 24 h and 72 h trajectory variables should be interpreted as pragmatic clinical time-window summaries rather than uniformly timed experimental measurements.
7. Median imputation was used as a conservative fold-wise strategy, but it does not fully preserve patient-specific physiological trajectories. LOCF and KNN imputation were not used because of sparse sampling, outcome-associated missingness, and the risk of instability or leakage in a modest cohort. Future studies should compare alternative longitudinal imputation strategies within nested validation frameworks.
8. Severity-proxy dominance: respiratory_support (maximum over 72 h) is a strong organ-support intensity marker and may encode treatment escalation decisions; therefore, respiratory_support-enriched models are best interpreted as time-updated severity-aware clinical-status models. The NoRS sensitivity analysis partially mitigates this concern by showing meaningful performance persists without that feature. For this reason, the primary interpretation should rely on models, excluding respiratory_support, whereas respiratory_support-enriched models should be viewed only as secondary severity-aware analyses.
9. Internal validation only: repeated cross-validation reduces optimism but does not replace external validation [20,21,23,25,26,27,28].
10. The primary endpoint was ICU death versus transfer to ward, which is a pragmatic but process-sensitive ICU-course outcome and represents a potential source of endpoint noise. It does not directly capture longer-term neurological recovery, hospital survival, or functional status and may partly reflect local discharge practices, bed availability, treatment-limitation decisions, or withdrawal/withholding of life-sustaining therapy.
11. Limited neurological and treatment-context variables are an important limitation. Although coma was operationally defined in the Methods, standardized admission Glasgow Coma Scale values, systematic etiologic categories of coma, sedative agent type, sedative dose and duration, neuromuscular-blocker use, targeted temperature management (TTM), admission-specific mechanical ventilation status, and total duration of invasive ventilation were not consistently available. These factors may influence neurological responsiveness, respiratory physiology, mechanical ventilation requirements, and the interpretation of ABG/SpO_2_ trajectories. Therefore, the present cohort should be interpreted as a pragmatic ICU population with documented coma or unresponsiveness, not as an etiologically homogeneous neurocritical-care cohort. The term “coma” is therefore used operationally in this study and should not be interpreted as a strictly defined neurocritical-care syndrome. Future studies should prospectively collect coma etiology, GCS, sedation and neuromuscular-blocker exposure, targeted temperature management (TTM), ventilation duration, and longer-term neurological outcomes. The outcome should therefore be interpreted as ICU-course disposition/mortality rather than true long-term neurological or functional prognosis.

### 4.8. Future Directions

A pragmatic next step is external validation in a second site, followed by prospective evaluation where predictions are computed in real time and compared against clinician judgement and decision pathways. Methodologically, future work should explore approaches that explicitly handle informative missingness and competing risks (early death vs. early discharge) [21,26,27,28]. Finally, extending from ICU mortality to longer-horizon endpoints (e.g., neurological recovery, functional outcome) [4,5,6] would align more directly with patient-centred coma prognostication.

## 5. Conclusions

In this single-centre cohort of 108 ICU patients with clinically documented coma or unresponsiveness, we developed and internally validated staged, explainable machine-learning models for ICU-course outcome using routine arterial blood gases, SpO_2_, and admission laboratory biomarkers measured at ICU admission and during the first 72 h. The models are best interpreted as exploratory, time-updated prognostic tools rather than admission-only predictors, particularly because respiratory_support summarized organ-support intensity over the first 72 h. In the main interpretive analyses, models incorporating early physiological trajectory features at 24 h and 72 h showed numerically higher discrimination than the earlier-stage model, but a definitive incremental prognostic value over parsimonious admission-only benchmarks was not established.

A prominent finding of this cohort was the extreme separation of outcomes by organ-support intensity: the derived respiratory_support variable, defined as the highest respiratory support level during the first 72 h, captured a strong severity gradient. This feature materially contributed to the very high discrimination observed in the respiratory_support-enriched models and indicates that organ-support intensity was a dominant prognostic signal in this cohort. Prespecified sensitivity analyses, excluding respiratory_support, showed that clinically meaningful discrimination persisted without this dominant severity proxy, particularly when trajectory information was available. However, complete-window sensitivity analyses and benchmark comparisons indicate that the trajectory-associated signal should be interpreted as exploratory and hypothesis-generating rather than as proof of independent incremental prognostic value.

To address key methodological vulnerabilities in modest ICU ML datasets, we implemented robustness checks aimed at leakage detection and severity-proxy dominance. Under label permutation, discrimination collapsed toward chance levels, supporting the absence of systematic pipeline leakage. Decision-curve analysis further suggested that time-updated models can provide net benefit over default strategies across clinically relevant thresholds, reinforcing that improvements in discrimination may translate into potential bedside utility.

Clinically, these findings support framing trajectory-based models as time-updated prognostic tools rather than admission-only predictors. These findings should not be interpreted as establishing a deployable ICU mortality score or as demonstrating superiority over SOFA/APACHE/SAPS; rather, they support the feasibility and physiological plausibility of ABG/SpO_2_ trajectory-based risk updating in a limited retrospective cohort. When interpreted in this way, explainable ML can complement bedside reasoning by highlighting patient-specific risk contributions, such as persistent hypoxemia, failure to improve oxygenation, or sustained inflammatory burden, using routinely available measurements.

Future work should focus on prospective, multicentre validation with standardized definitions of coma and organ support, explicit handling of outcome-associated missingness, benchmarking against established ICU severity scores when component variables are available, and evaluation in real-time workflows to determine whether trajectory-based explainable models can improve consistency of prognostic communication and decision-making without contributing to self-fulfilling prophecy effects. Because of the modest sample size and internal-validation-only design, comparisons between logistic regression, random forest, and XGBoost should be considered exploratory rather than evidence of algorithmic superiority.

### Clinical Implications

This work supports a pragmatic, bedside-aligned use case: updating prognostic estimates at predefined clinical reassessment points, such as 24 h and 72 h, using routinely collected ABG/SpO_2_, admission biomarkers, and derived early trajectory features. In practice, the model output can be treated as an additional trajectory summary to complement neurological examination and multidisciplinary discussion, particularly to highlight failure to improve oxygenation/acid–base status or persistently high inflammatory burden. Because organ-support intensity strongly influences risk, the model should be interpreted in context as a time-updated severity-aware tool, and sensitivity results, excluding respiratory_support, provide reassurance that meaningful prognostic signal remains even when this dominant severity proxy is removed.

## Figures and Tables

**Table 1:** Baseline demographic, clinical, and laboratory characteristics of the study population (n = 108). Continuous variables are presented as mean ± standard deviation or median (interquartile range) depending on distribution, while categorical variables are presented as counts and percentages.

Variable	Overall Cohort (n = 108)
Age, years	68.9 ± 14.4
Male sex	62 (57.4%)
Comorbidity score	2 (IQR 2–3)
COVID-19 positive	55 (50.9%)
SpO_2_ at ICU admission (%)	85 (IQR 78–89.3)
Arterial pH at admission	7.37 ± 0.11
PaCO_2_ at admission (mmHg)	32.8 (IQR 28.9–37.6)
PaO_2_ at admission (mmHg)	58.8 (IQR 52.5–72.2)
Bicarbonate at admission (mmol/L)	21.8 ± 6.5
Leukocytes (×10^9^/L)	11.2 (IQR 8.0–15.7)
C-reactive protein (mg/L)	96 (IQR 54.8–152.1)
Procalcitonin (ng/mL)	0.70 (IQR 0.14–2.54)
Lactate dehydrogenase (U/L)	1151 (IQR 747–1866.8)
D-dimer	3.5 (IQR 2.8–4.3)
INR	1.30 (IQR 1.12–1.62)
aPTT (s)	32.8 (IQR 26.8–39.3)
Respiratory support level	2 (IQR 1–2)

**Table 2:** Comparison of demographic, physiological, and laboratory variables between ICU survivors and non-survivors. Continuous variables are presented as median [interquartile range], while categorical variables are presented as counts and percentages. Continuous variables were compared using the Mann–Whitney U test, while categorical variables were compared using the chi-square test or Fisher’s exact test, as appropriate.

Variable	Survivors (n = 37)	Deaths (n = 71)
Male sex	17/37 (45.9%)	45/71 (63.4%)
Age, years	72 [IQR 18]	72 [IQR 16]
Comorbidity score	2 [IQR 1]	2 [IQR 1]
COVID-19 positive	16/37 (43.2%)	39/71 (54.9%)
SpO_2_ at admission (%)	87 [IQR 17]	85 [IQR 10]
Arterial pH at admission	7.41 [IQR 0.07]	7.35 [IQR 0.13]
PaCO_2_ at admission (mmHg)	32.5 [IQR 6.8]	34 [IQR 10.1]
PaO_2_ at admission (mmHg)	69.2 [IQR 32.7]	54 [IQR 15.6]
Bicarbonate at admission	23 [IQR 9]	22 [IQR 8.5]
SpO_2_ at 24 h (%)	95 [IQR 6]	92 [IQR 5]
Arterial pH at 24 h	7.42 [IQR 0.08]	7.35 [IQR 0.11]
PaCO_2_ at 24 h (mmHg)	32.2 [IQR 6]	36 [IQR 10.2]
PaO_2_ at 24 h (mmHg)	87 [IQR 30.6]	56.3 [IQR 11.9]
SpO_2_ at 72 h (%)	97 [IQR 3.25]	92 [IQR 6]
Arterial pH at 72 h	7.46 [IQR 0.06]	7.35 [IQR 0.08]
PaCO_2_ at 72 h (mmHg)	33.7 [IQR 9.47]	36.5 [IQR 13.4]
PaO_2_ at 72 h (mmHg)	78.9 [IQR 24.4]	54.5 [IQR 12.2]
Leukocytes	9.12 [IQR 7.39]	12 [IQR 8.08]
C-reactive protein	71.5 [IQR 77.5]	114 [IQR 96.9]
Procalcitonin	0.325 [IQR 1.75]	0.8 [IQR 2.54]
Lactate dehydrogenase	741 [IQR 521]	1570 [IQR 1380]
D-dimer	3.2 [IQR 1.1]	3.6 [IQR 1.5]
INR	1.21 [IQR 0.34]	1.35 [IQR 0.46]
aPTT	28.4 [IQR 11.2]	33.9 [IQR 13.6]
Respiratory support severity (0/1/2), n (%)	0: 22/37 (59.5%); 1: 11/37 (29.7%); 2: 4/37 (10.8%)	0: 0/71 (0.0%); 1: 1/71 (1.4%); 2: 70/71 (98.6%)

**Table 3:** Primary NoRS logistic-regression model performance for ICU mortality. Performance metrics were estimated using repeated stratified cross-validation with aligned out-of-fold predictions. These models exclude respiratory_support and represent the main interpretive analyses. AUC-ROC confidence intervals were estimated by non-parametric bootstrap resampling of aligned out-of-fold predicted probabilities.

Model	AUC-ROC	95% CI	PR-AUC	Brier Score	Accuracy	Sensitivity	Specificity	Precision	F1
Model A_noRS	0.822	0.739–0.897	0.889	0.167	0.759	0.775	0.730	0.846	0.809
Model B_noRS	0.848	0.766–0.919	0.909	0.155	0.759	0.704	0.865	0.909	0.794
Model C_noRS	0.895	0.830–0.951	0.942	0.126	0.815	0.775	0.892	0.932	0.846

**Note:** Bootstrap 95% confidence intervals for AUC-ROC were computed at the patient level from aligned out-of-fold predicted probabilities.

**Table 4:** Operating-point confusion matrices for Model C using aligned out-of-fold predictions and the Youden-index threshold. The respiratory_support-enriched models include the 72 h support-intensity summary, whereas NoRS models exclude respiratory_support.

Model C Implementation	Observed Outcome	Predicted ICU Death	Predicted Ward Transfer
Logistic regression, respiratory_support-enriched	ICU death	66	5
Logistic regression, respiratory_support-enriched	Ward transfer	0	37
Random forest, respiratory_support-enriched	ICU death	66	5
Random forest, respiratory_support-enriched	Ward transfer	0	37
Logistic regression, NoRS	ICU death	55	16
Logistic regression, NoRS	Ward transfer	4	33
Random forest, NoRS	ICU death	63	8
Random forest, NoRS	Ward transfer	8	29

**Table 5:** Positioning of the present study relative to common approaches in ICU prognostication.

Approach	Typical Inputs	Time Handling	Strengths	Common Limitations	Contribution of the Present Study
Traditional ICU severity scores	Vitals/labs, organ failure, sometimes worst values	Often static snapshot or “worst in window”	Fast, familiar, widely validated	Limited trajectory interpretability; not coma-focused	Explicit 24–72 h trajectory framing using bedside ABG/SpO_2_ variables
Large-scale ICU ML (EHR-based)	High-dimensional EHR, frequent sampling	Explicit time-series models or engineered windows	Strong performance in large cohorts	Often black-box; hard to operationalize outside EHR-rich settings	Uses routine ABG/SpO_2_ + biomarkers; interpretable explanations
Neurocritical-care ML	EEG/imaging/multimodal monitoring	Variable	Etiology-specific performance gains	Requires specialised modalities; limited generalizability	Heterogeneous real-world coma; routine data only
Present study	ABG/SpO_2_ at 0/24/72 h + biomarkers + organ-support index	Explicit staged time-updated prognosis	Transparent, bedside-aligned explainability; DCA	Single centre; missing follow-up; strong severity proxy	NoRS + label-permutation robustness; interpretable trajectory phenotypes

## Data Availability

The data presented in this study are not publicly available because they contain sensitive patient information and are subject to privacy, ethical, and institutional restrictions. De-identified data may be available from the corresponding author upon reasonable request and with permission of the relevant institution and ethics requirements.

## Supplementary Materials

The following supporting information can be downloaded at: https://www.mdpi.com/article/10.3390/jcm15135056/s1.
