# Supplementary material for: Time-Updated Prognostic Modeling in ICU Patients with Documented Coma or Unresponsiveness Using Routine Arterial Blood Gas Trajectories: An Exploratory Explainable Machine-Learning Study
Source: J Clin Med. 2026 Jun 29;15(13):5056. doi: 10.3390/jcm15135056 (PMC13362702; doi:10.3390/jcm15135056)

Supplementary Materials

Supplementary Methods

S1. Out-of-fold (OOF) prediction framework

Performance estimates were obtained using repeated stratified k-fold cross-validation. For each split, models were trained on the training folds and probabilistic predictions were generated only for patients in the held-out fold. When repeated cross-validation was used, each patient could receive multiple held-out predictions across repetitions. These predictions were averaged to obtain a single aligned out-of-fold probability per patient. This approach ensures that every prediction used for evaluation is generated from a model that did not see that patient during training, enabling unbiased downstream calibration assessment, decision-curve analysis, and explainability analyses.

S2. Decision curve analysis (DCA)

Clinical utility was evaluated using decision curve analysis based on aligned OOF probabilities. Net benefit was computed across a range of threshold probabilities and compared with two default strategies: treating all patients and treating none. This approach evaluates whether the predictive models provide clinically meaningful decision support across plausible risk thresholds.

S3. Gradient boosting (XGBoost)

In addition to logistic regression and random forests, gradient boosting models were implemented using the XGBoost framework. Models were trained using the same predictor sets and the same cross-validation and OOF prediction pipeline to ensure consistent and comparable performance estimation.

S4. Explainability

Global feature contributions were quantified using SHAP (Shapley additive explanations) values. To further characterize the direction and potential nonlinearity of predictor effects, partial dependence plots (PDPs) were generated for the highest-ranked variables and selected trajectory features. These analyses provide interpretable insight into how physiological and biomarker variables influence predicted mortality risk.

S5. Robustness and leakage-control analyses

Two additional analyses were performed to assess model robustness and potential pipeline leakage. First, to evaluate sensitivity to dominance by severity proxies, all models were re-trained after removing the respiratory_support variable (NoRS analysis). Second, a label-permutation sanity check was conducted by randomly shuffling ICU outcome labels and repeating the full modeling pipeline under identical settings. Under this condition, model discrimination is expected to collapse toward chance levels if no information leakage is present.

Table S1. Performance of logistic regression and random forest models after excluding respiratory_support (NoRS analysis).

Model	Algorithm	AUC_ROC	PR_AUC	Brier	Accuracy	Sensitivity	Specificity	Precision	F1
Model_A_noRS	LogReg	0.822	0.889	0.167	0.759	0.775	0.73	0.846	0.809
Model_B_noRS	LogReg	0.848	0.909	0.155	0.759	0.704	0.865	0.909	0.794
Model_C_noRS	LogReg	0.895	0.942	0.126	0.815	0.775	0.892	0.932	0.846
Model_A_noRS	RandomForest	0.841	0.897	0.15	0.833	0.915	0.676	0.844	0.878
Model_B_noRS	RandomForest	0.852	0.899	0.147	0.852	0.915	0.73	0.867	0.89
Model_C_noRS	RandomForest	0.891	0.923	0.131	0.852	0.887	0.784	0.887	0.887

Values are based on aligned out-of-fold (OOF) predictions obtained by repeated stratified cross-validation.

Table S2. Gradient boosting (XGBoost) performance for the main staged models using aligned OOF predictions.

Model	Algorithm	AUC_ROC	PR_AUC	Brier	Accuracy	Sensitivity	Specificity	Precision	F1	Threshold_Youden
Model_A	XGBoost	0.987	0.994	0.033	0.963	0.958	0.973	0.986	0.971	0.763
Model_B	XGBoost	0.987	0.995	0.032	0.972	0.972	0.973	0.986	0.979	0.732
Model_C	XGBoost	0.988	0.995	0.033	0.963	0.958	0.973	0.986	0.971	0.793

Threshold_Youden denotes the operating threshold that maximized Youden's J on aligned OOF predictions.

Table S3. Gradient boosting (XGBoost) performance after excluding respiratory_support (NoRS analysis).

Model	Algorithm	AUC_ROC	PR_AUC	Brier	Accuracy	Sensitivity	Specificity	Precision	F1	Threshold_Youden
Model_A_noRS	XGBoost_NoRS	0.83	0.898	0.156	0.824	0.901	0.676	0.842	0.871	0.529
Model_B_noRS	XGBoost_NoRS	0.83	0.889	0.148	0.833	0.901	0.703	0.853	0.877	0.592
Model_C_noRS	XGBoost_NoRS	0.861	0.906	0.128	0.861	0.958	0.676	0.85	0.901	0.448

Table S4. Univariate associations between clinical variables and ICU mortality. Continuous variables were analyzed using Student's t-test or Mann–Whitney U test depending on distribution, while categorical variables were analyzed using chi-square or Fisher's exact tests. False discovery rate (FDR) correction was applied to account for multiple testing.Effect sizes are reported as Cohen's d for continuous variables, the φ coefficient for 2×2 chi-square tests, Cramér's V for larger contingency tables, and odds ratios for Fisher's exact tests.

Variable	Test	Effect size	p-value	FDR-adjusted p	Significant after FDR
Respiratory support	Chi²	0.90	<0.001	<0.001	Yes
PaO_2 at 24h	Mann–Whitney	−1.78	<0.001	<0.001	Yes
PaO_2 at 72h	Mann–Whitney	−2.37	<0.001	<0.001	Yes
LDH	Mann–Whitney	1.02	<0.001	<0.001	Yes
PaO_2 at admission	Mann–Whitney	−1.14	<0.001	<0.001	Yes
pH at 72h	Mann–Whitney	−1.39	<0.001	<0.001	Yes
SpO_2 at 72h	Mann–Whitney	−1.07	<0.001	0.001	Yes
pH at 24h	Mann–Whitney	−0.87	<0.001	0.001	Yes
SpO_2 at 24h	Mann–Whitney	−0.75	<0.001	0.004	Yes
ΔPaO_2 72h	Mann–Whitney	−0.52	0.002	0.009	Yes
ΔPaO_2 24h	Mann–Whitney	−0.54	0.003	0.012	Yes
CRP	Mann–Whitney	0.61	0.0046	0.014	Yes
D-dimer	Mann–	0.62	0.0048	0.014	Yes

Variable	Test	Effect size	p-value	FDR-adjusted p	Significant after FDR
	Whitney				
Availability of 72h ABG	Chi²	0.26	0.006	0.017	Yes
Bicarbonate at 72h	Welch t-test	−0.78	0.0075	0.020	Yes
aPTT	Mann–Whitney	0.46	0.0087	0.021	Yes
$PaCO_2$ at 24h	Mann–Whitney	0.47	0.013	0.031	Yes
Leukocytes	Mann–Whitney	0.22	0.021	0.045	Yes

Supplementary Table S5. Clinically relevant variables unavailable in the retrospective dataset

Variable/domain	Availability in current dataset	Consequence for interpretation
Glasgow Coma Scale	Not consistently available	Coma could not be defined using GCS threshold; operational clinical definition used
Etiology of coma	Not consistently available	No subgroup analysis by post-anoxic, stroke, TBI, metabolic, septic, or toxic coma
Sedation / neuromuscular blockade	Not consistently available	Cannot distinguish all neurological coma from pharmacological unresponsiveness
Targeted temperature management	Not consistently available	Cannot adjust for TTM in possible post-cardiac-arrest cases
FiO_2	Not available	PaO_2/FiO_2 and respiratory SOFA could not be calculated
Mechanical ventilation duration	Not consistently available	respiratory_support interpreted only as 72 h support-intensity summary
Vasopressors / MAP	Not available	Cardiovascular SOFA/APACHE/SAPS components unavailable
Creatinine / urine output	Not available	Renal SOFA/APACHE/SAPS components unavailable
Bilirubin / platelets	Not available	Liver and coagulation SOFA components unavailable
Sodium / potassium / hematocrit	Not available	APACHE II/SAPS II could not be reconstructed

Variable/domain	Availability in current dataset	Consequence for interpretation
Hospital mortality / neurological outcome	Not available	Endpoint limited to ICU death versus ward transfer

Supplementary Table S6. Secondary respiratory_support-enriched severity-aware models. Because respiratory_support was defined as the highest respiratory-support level during the first 72 h, these models are interpreted as secondary severity-aware clinical-status analyses and not as strict admission-only prediction models.

Model	Algorithm	AUC-ROC	PR-AUC	Brier score	Accuracy	Sensitivity	Specificity	Precision	F1 score
Model A, secondary severity-aware version	Logistic regression	0.988	0.993	0.039	0.954	0.958	0.946	0.971	0.965
Model B (24 h trajectory features)	Logistic regression	0.992	0.996	0.036	0.954	0.944	0.973	0.985	0.964
Model C (72 h trajectory features)	Logistic regression	0.993	0.996	0.035	0.954	0.930	1.000	1.000	0.964
Model A, secondary severity-aware version	Random forest	0.986	0.994	0.058	0.972	0.986	0.946	0.972	0.979
Model B (24 h trajectory features)	Random forest	0.993	0.997	0.064	0.972	0.986	0.946	0.972	0.979
Model C (72 h trajectory features)	Random forest	0.994	0.997	0.059	0.954	0.930	1.000	1.000	0.964

Supplementary Table S7. Sensitivity analysis including ABG measurement-availability indicators.

Model	Added indicator	AUC-ROC	95% CI	PR-AUC	Brier
Model B_noRS LR	has_24h_abg	0.845	0.765–0.916	0.906	0.151
Model C_noRS LR	has_72h_abg	0.907	0.847–0.961	0.951	0.112
Model C_noRS LR	has_24h_abg + has_72h_abg	0.901	0.836–0.958	0.945	0.114

Supplementary Table S8. Simple benchmark model performance.

Model	AUC-ROC	95% CI	PR-AUC	Brier
Admission PaO_2 alone	0.788	0.698–0.871	0.865	0.185
Admission PaO_2 + LDH + CRP	0.862	0.787–0.929	0.920	0.150
Model A_noRS LR	0.822	0.739–0.897	0.889	0.167
Model C_noRS LR	0.895	0.830–0.951	0.942	0.126

Supplementary Table S9. Complete-window trajectory sensitivity analyses restricted to patients with recorded follow-up ABG measurements. Models were evaluated using the same repeated cross-validation and aligned out-of-fold prediction framework as the main analyses.

Analysis	N	ICU deaths	Survivors	Predictors	AUC-ROC	95% CI	PR-AUC	Brier	Accuracy	Sensitivity	Specificity
24 h ABG-available subgroup: Model A_noRS LR	82	57	25	16	0.857	0.751–0.937	0.926	0.145	0.817	0.860	0.720
24 h ABG-available subgroup: Model B_noRS	82	57	25	21	0.879	0.776–0.956	0.933	0.122	0.866	0.912	0.760

Analysis	N	ICU deaths	Survivors	Predictors	AUC-ROC	95% CI	PR-AUC	Brier	Accuracy	Sensitivity	Specificity
LR											
72 h ABG-available subgroup: Model A_noRS LR	66	50	16	16	0.759	0.602–0.888	0.901	0.175	0.788	0.860	0.563
72 h ABG-available subgroup: Model C_noRS LR	66	50	16	21	0.940	0.876–0.984	0.981	0.087	0.879	0.880	0.875

Note: The 24 h complete-window comparison evaluates Model A_noRS versus Model B_noRS among patients with available 24 h ABG measurements. The 72 h complete-window comparison evaluates Model A_noRS versus Model C_noRS among patients with available 72 h ABG measurements. These analyses are exploratory because subgroup sizes are smaller and outcome distributions are imbalanced.

Supplementary Table S10. Riley-framework-informed sample-size adequacy assessment for staged NoRS logistic-regression models.

Model	Observed N	ICU deaths	Survivors	Outcome prevalence	Candidate predictors	Observed C-statistic	Events per predictor	Non-events per predictor	Approx. required total N	Cohort meets requirement
Model A_noRS	108	71	37	0.657	16	0.822	4.44	2.31	935	No
Model B_noRS	108	71	37	0.657	21	0.848	3.38	1.76	1226	No
Model C_noRS	108	71	37	0.657	21	0.895	3.38	1.76	1226	No

Supplementary Figures

Figure S1. Label-permutation sanity-check ROC curve for Model A (logistic regression).

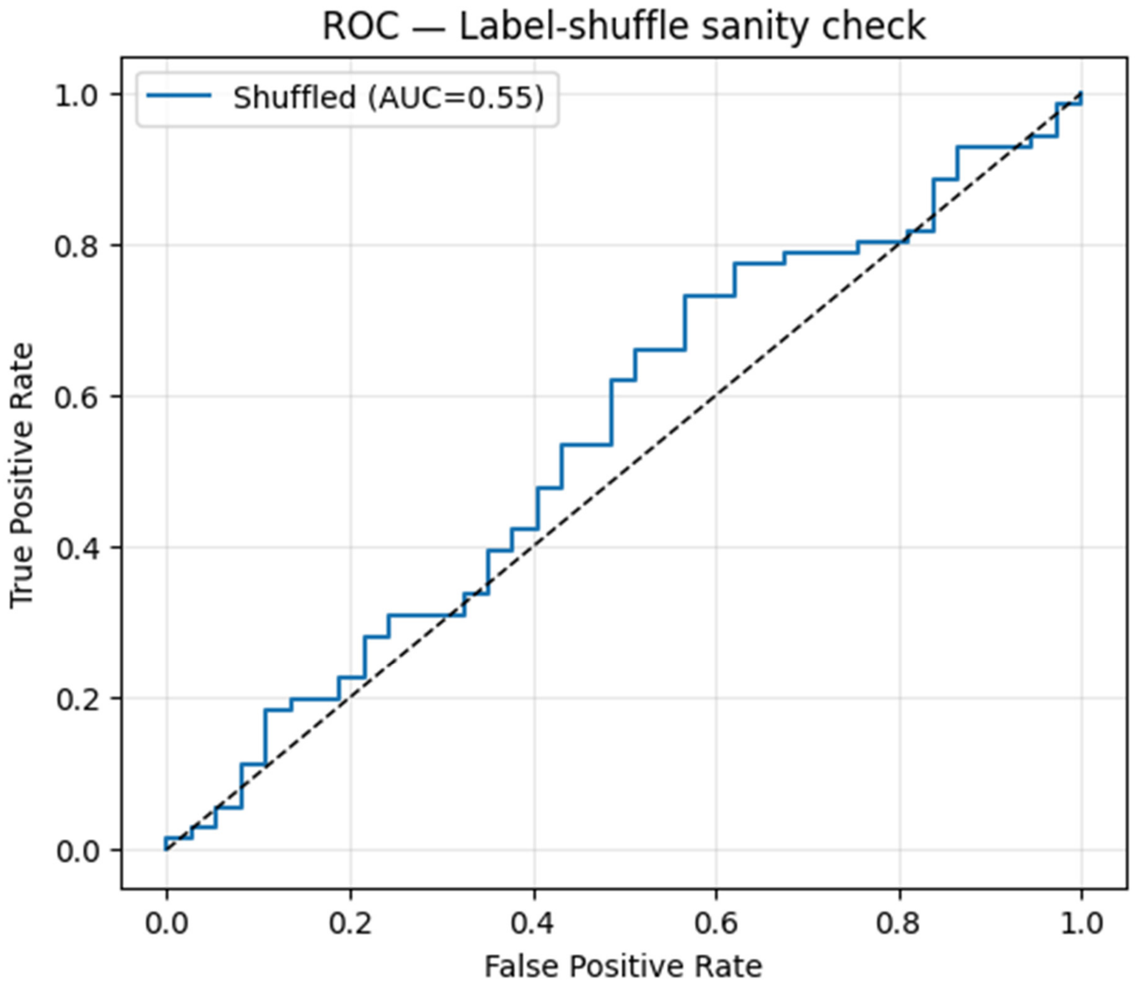

Figure S2. SHAP summary plot for Model C (XGBoost).

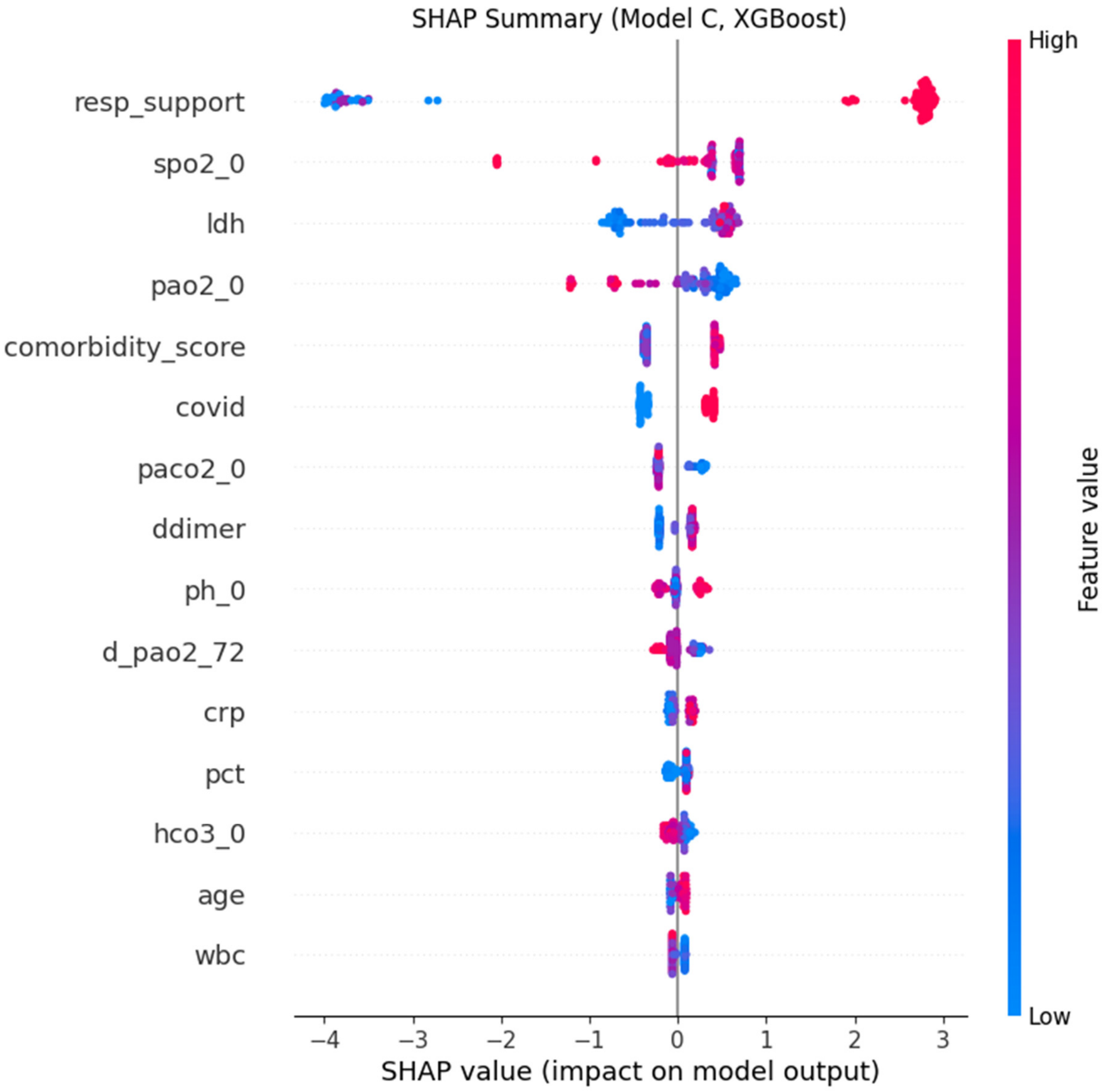

Figure S3. SHAP global importance for Model C (XGBoost).

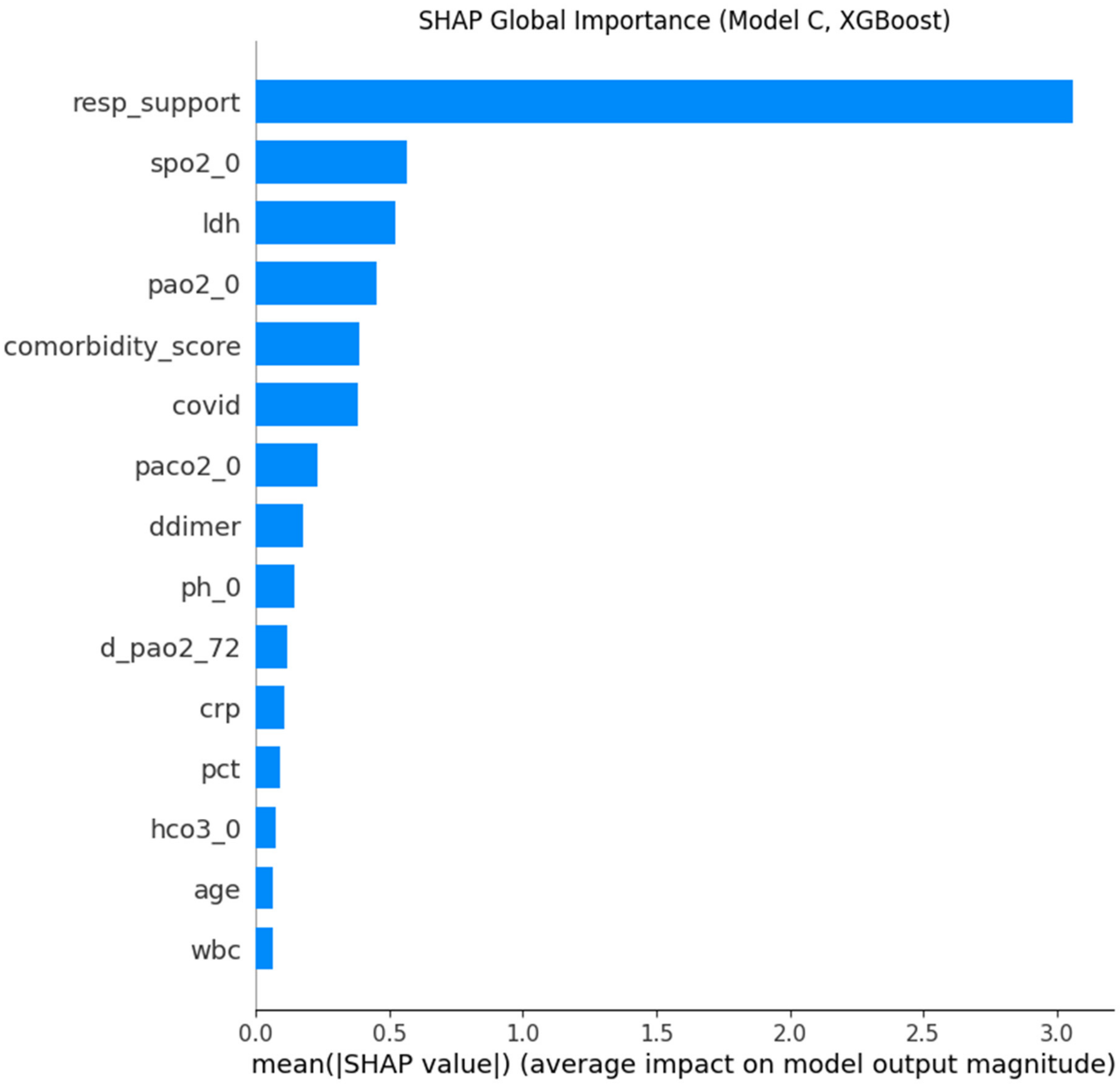

Figure S4. SHAP dependence plot for respiratory_support in Model C (XGBoost), colored by admission PaO2.

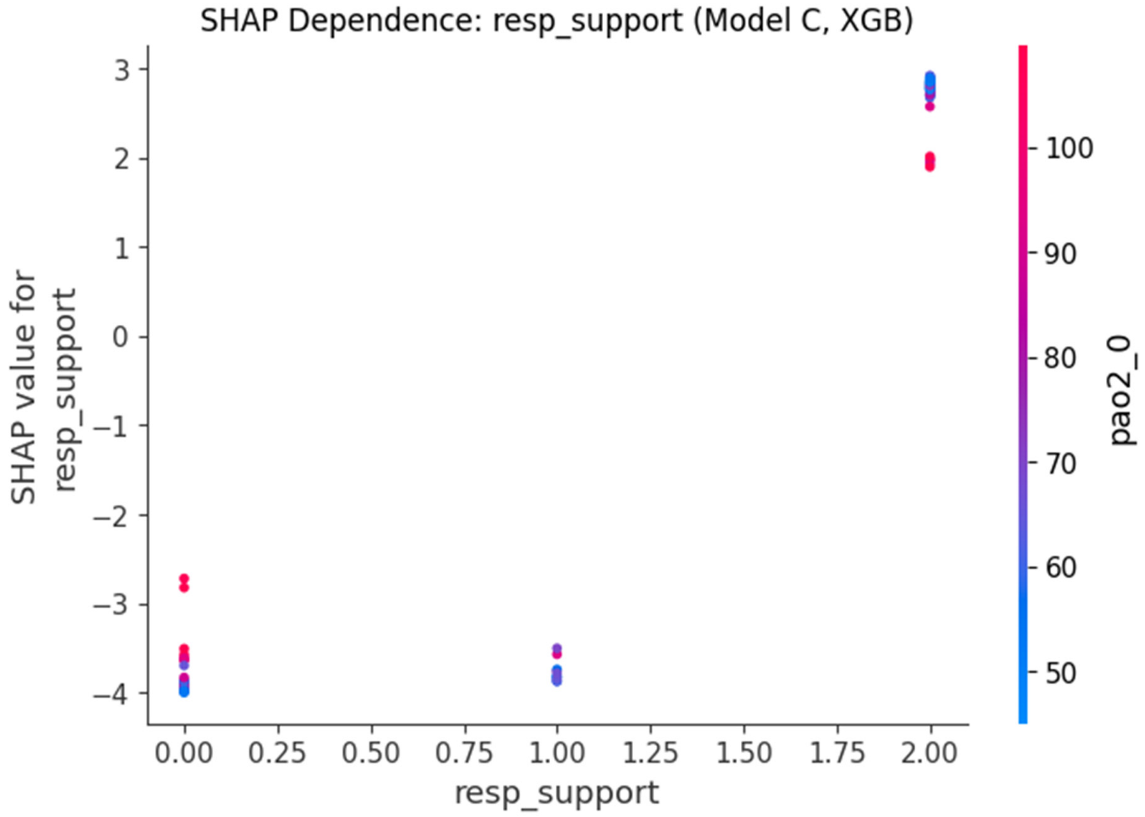

Figure S5. SHAP dependence plot for admission SpO2 in Model C (XGBoost), colored by respiratory_support.

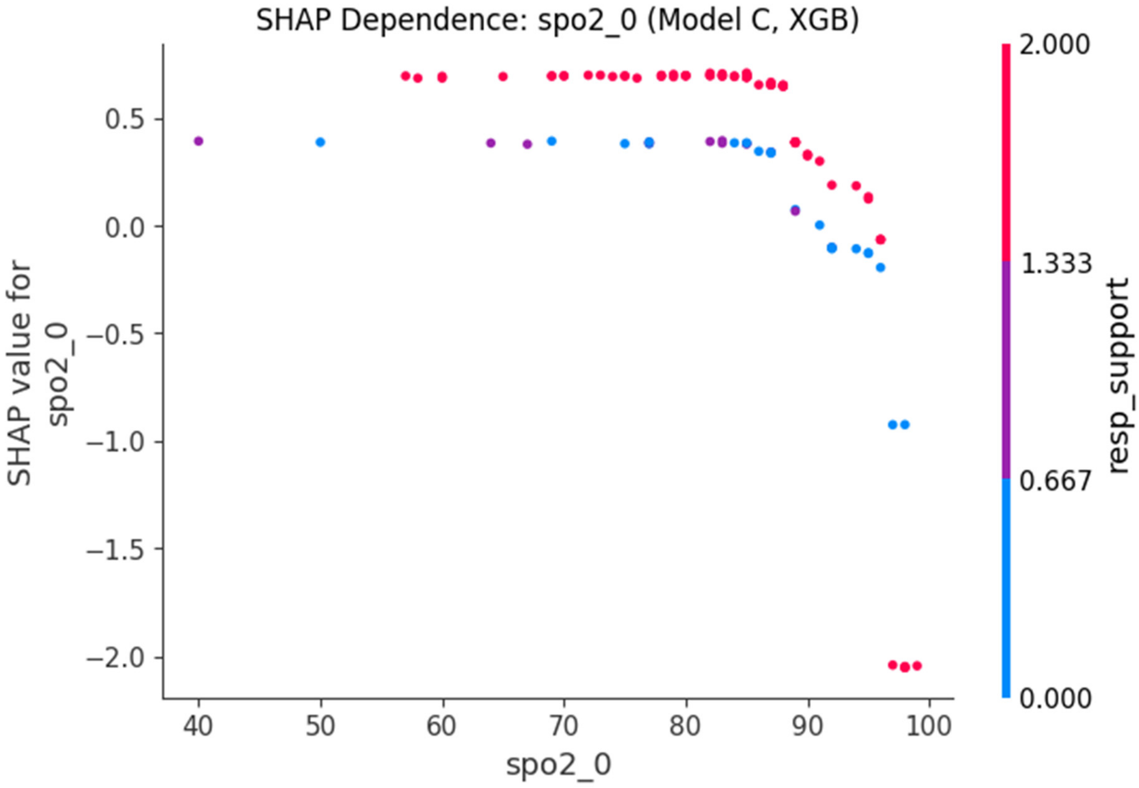

Figure S6. SHAP dependence plot for LDH in Model C (XGBoost), colored by admission PaO2.

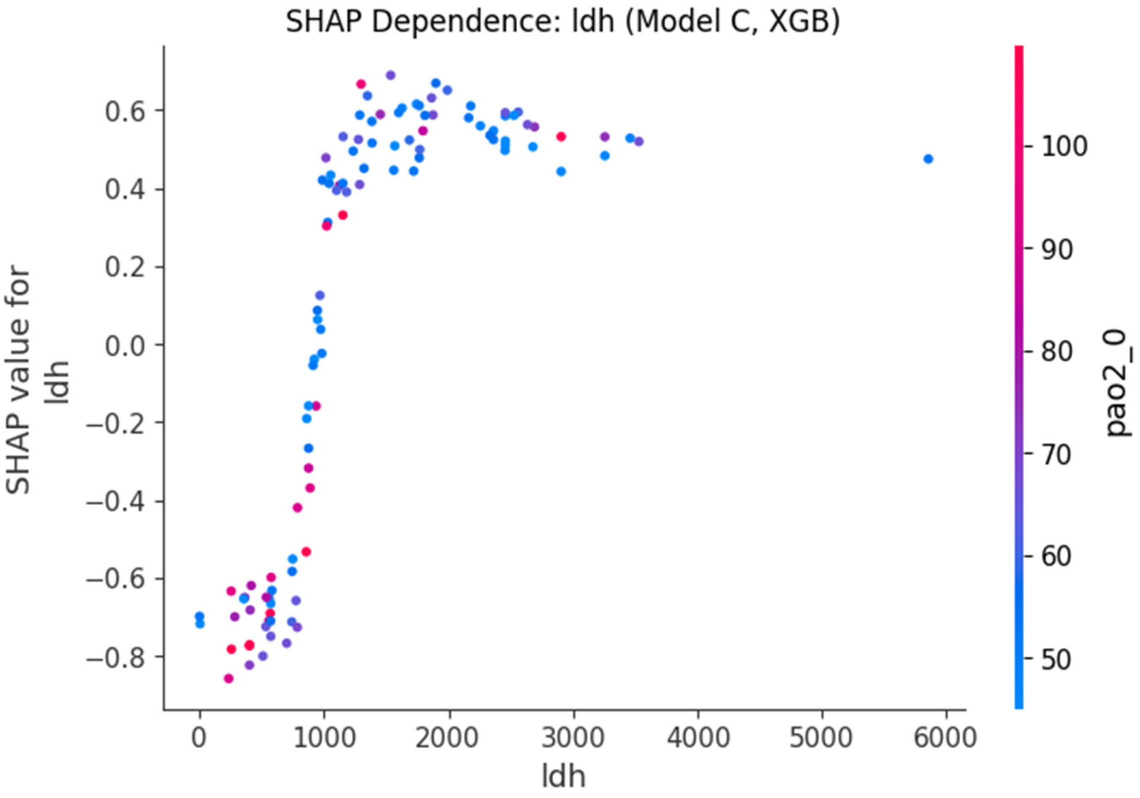

Figure S7. Partial dependence plot for respiratory_support in Model C (XGBoost).

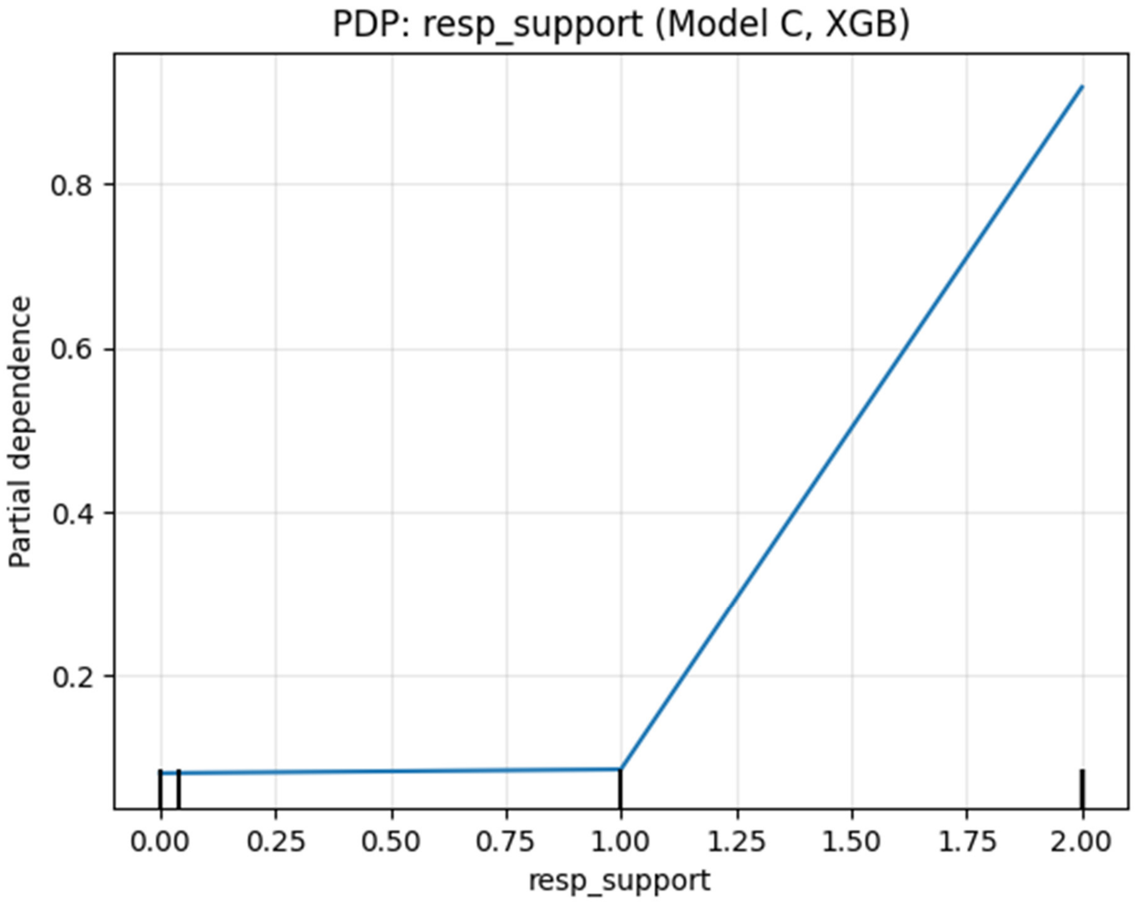

Figure S8. Partial dependence plot for admission SpO2 in Model C (XGBoost).

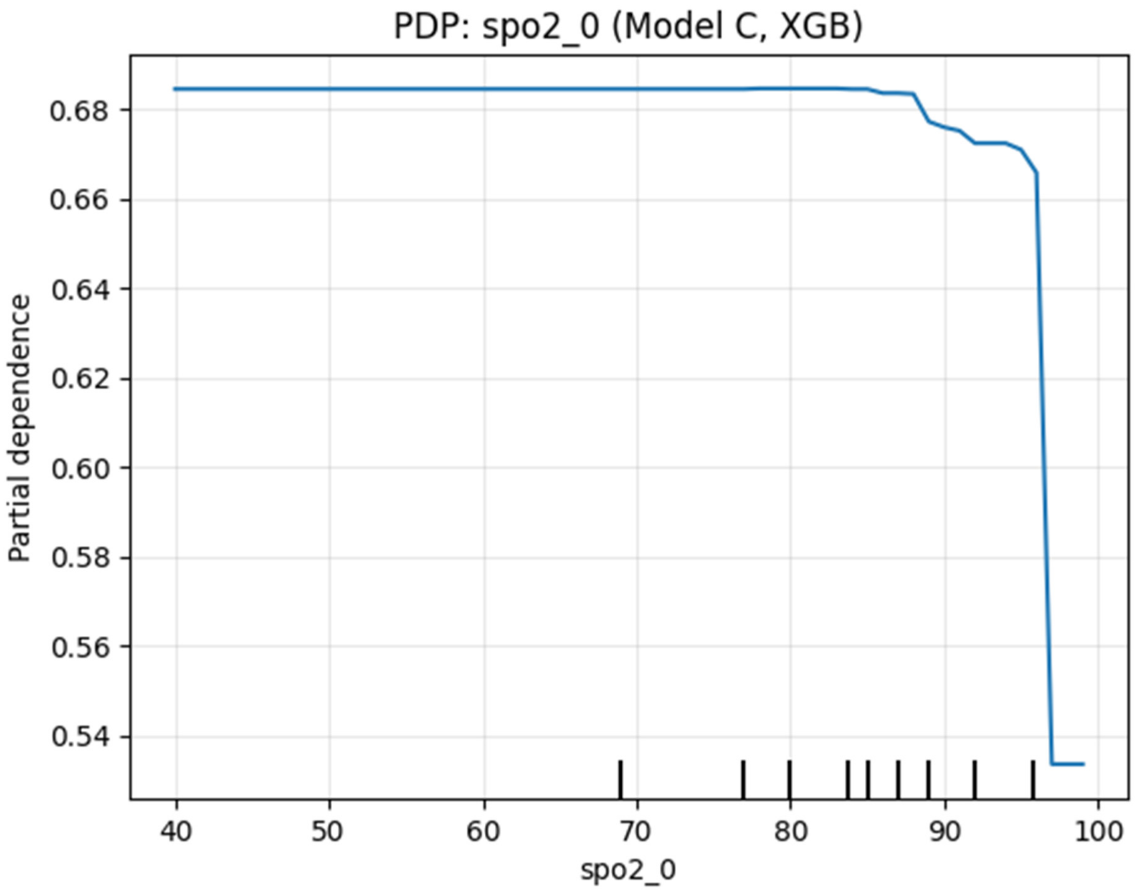

Figure S9. Partial dependence plot for LDH in Model C (XGBoost).

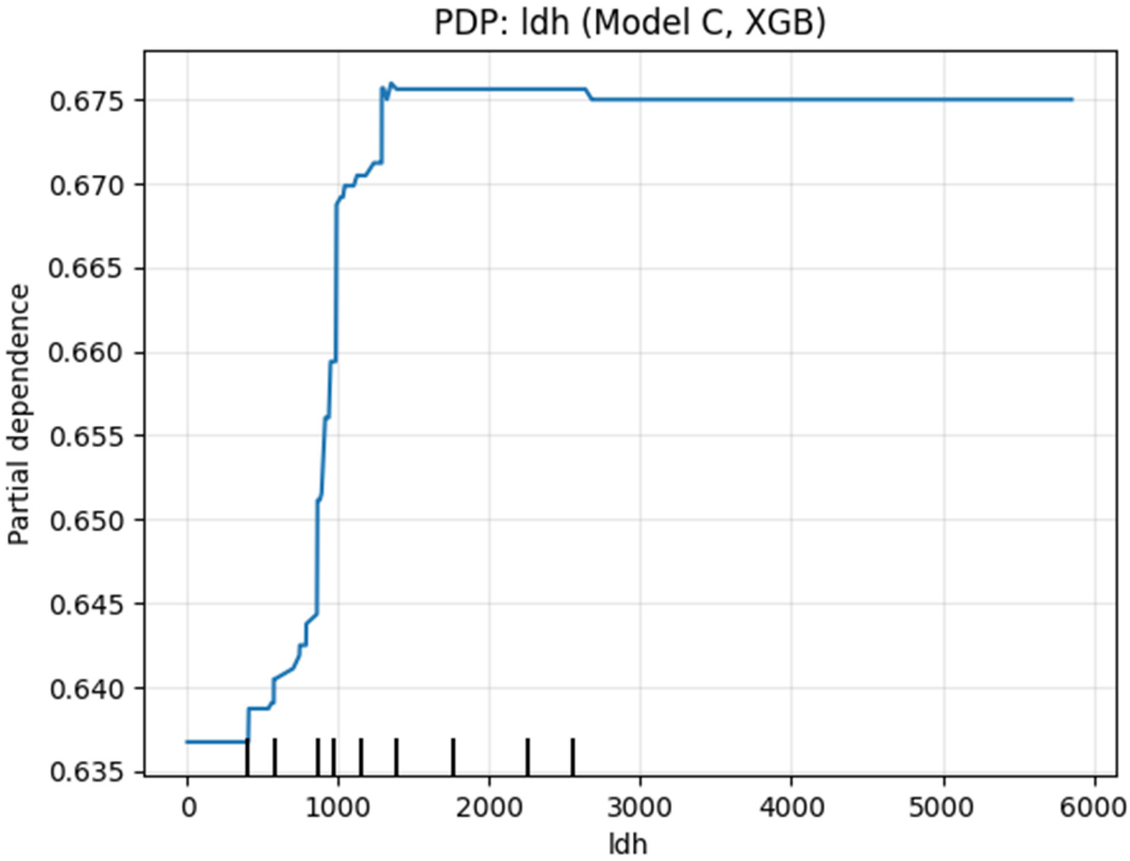

Figure S10. SHAP summary plot for Model C after excluding respiratory_support (XGBoost NoRS).

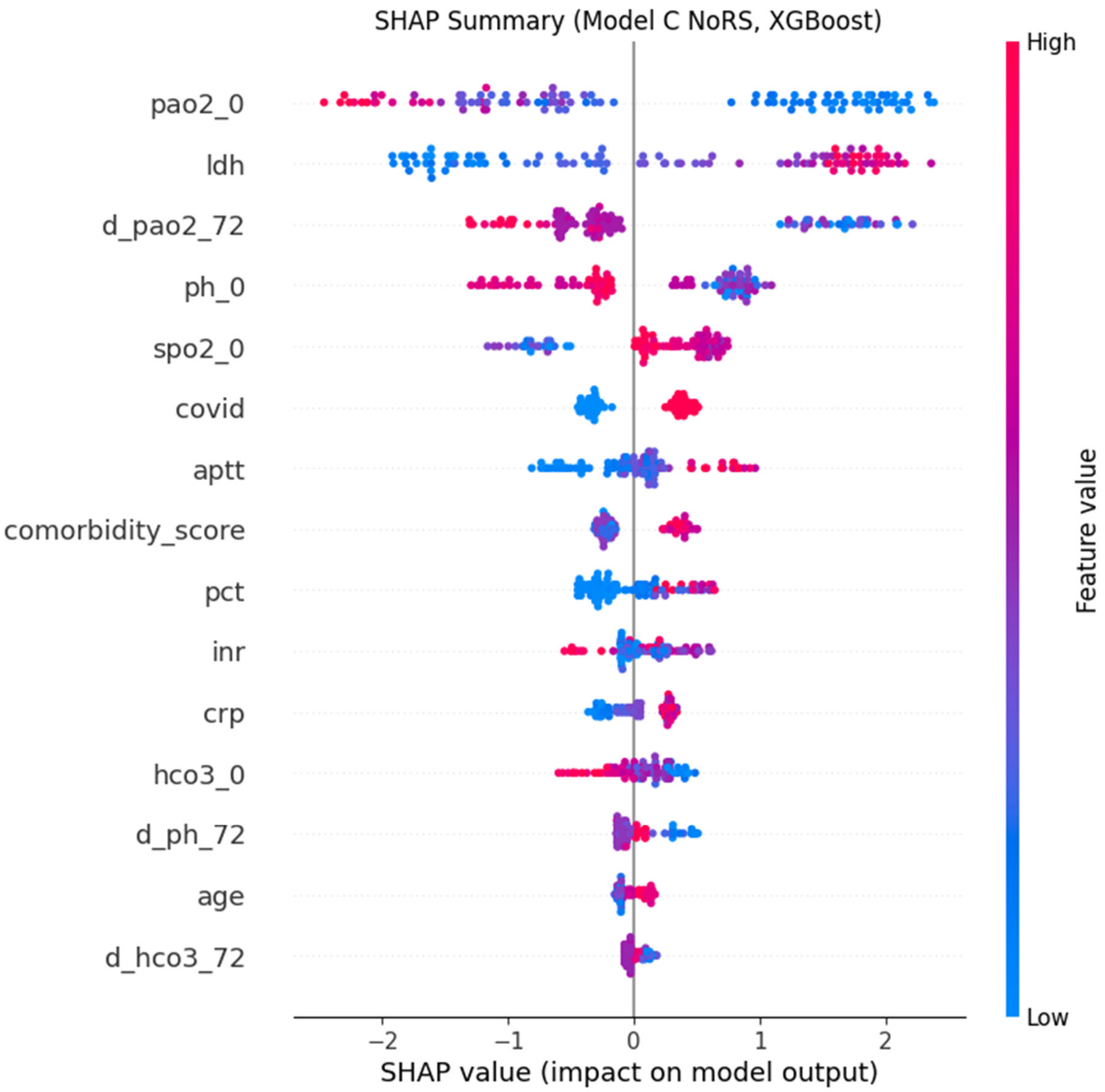

Figure S11. SHAP global importance for Model C after excluding respiratory_support (XGBoost NoRS).

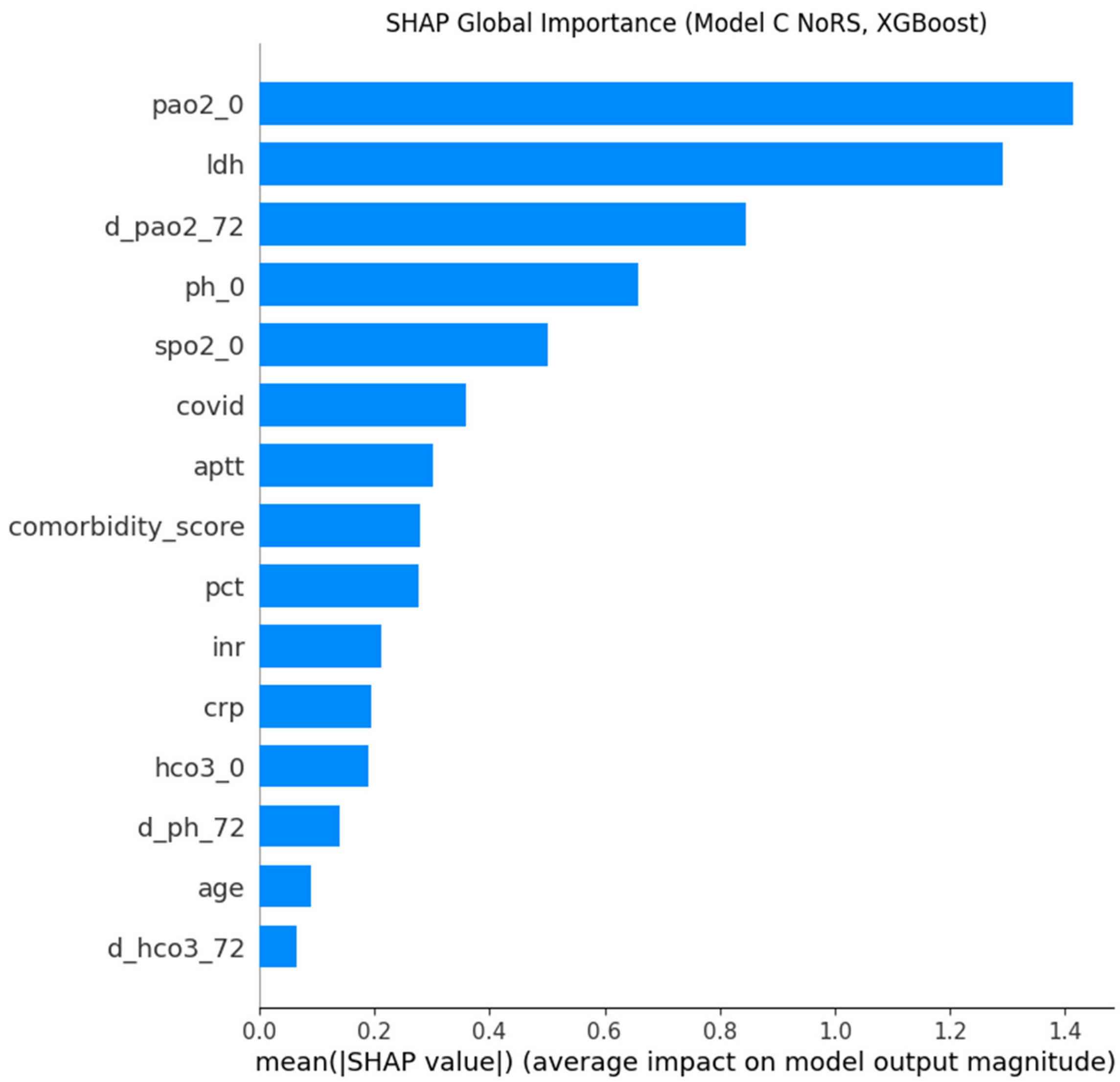

Figure S12. Partial dependence plot for admission PaO2 in Model C NoRS (XGBoost).

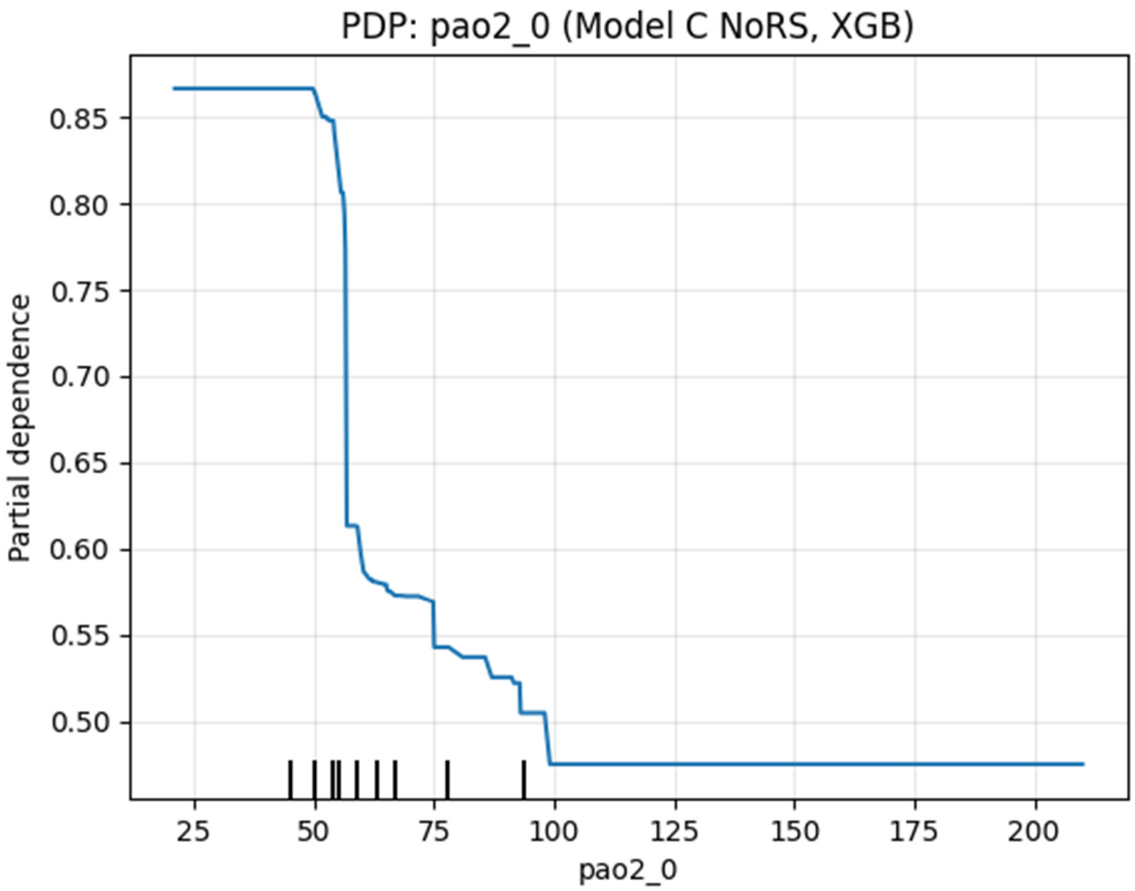

Figure S13. Partial dependence plot for LDH in Model C NoRS (XGBoost).

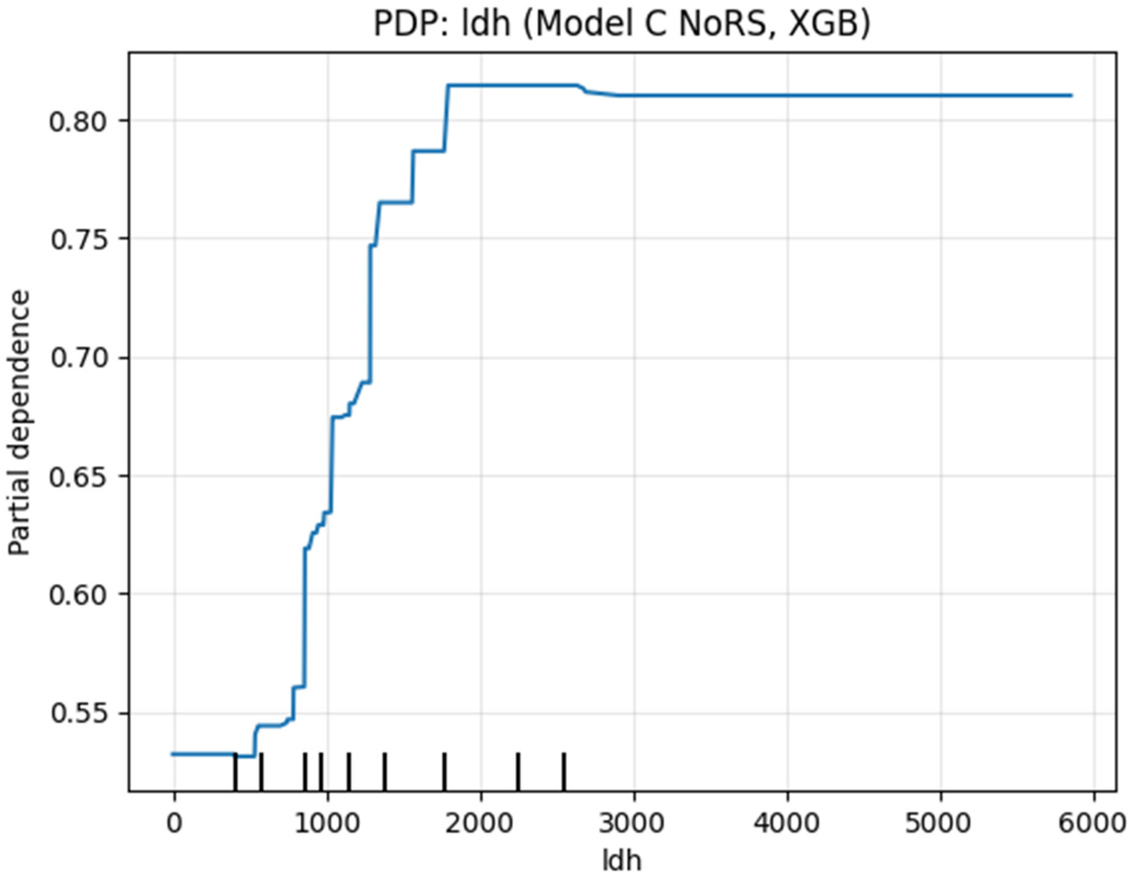

Figure S14. Partial dependence plot for delta PaO2 at 72 h in Model C NoRS (XGBoost).

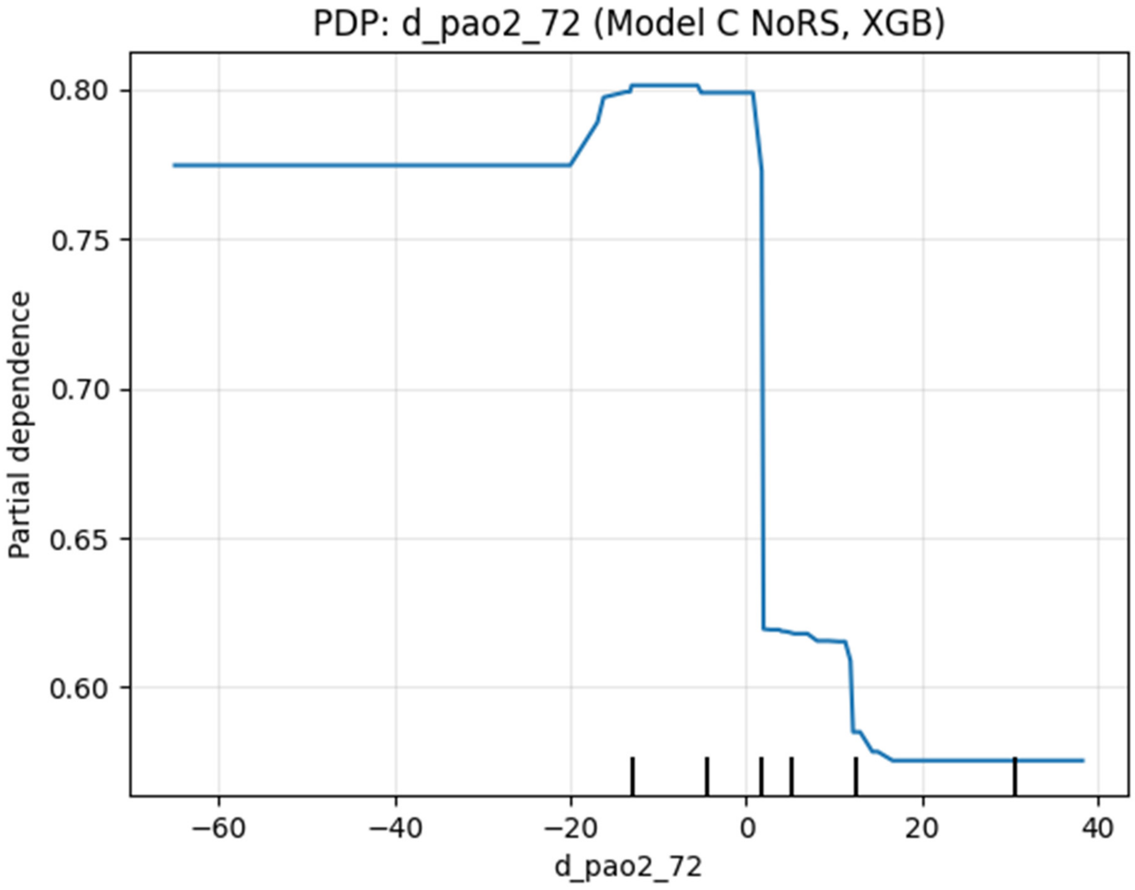

Figure S15. Decision curve analysis for NoRS XGBoost models based on aligned OOF predictions.

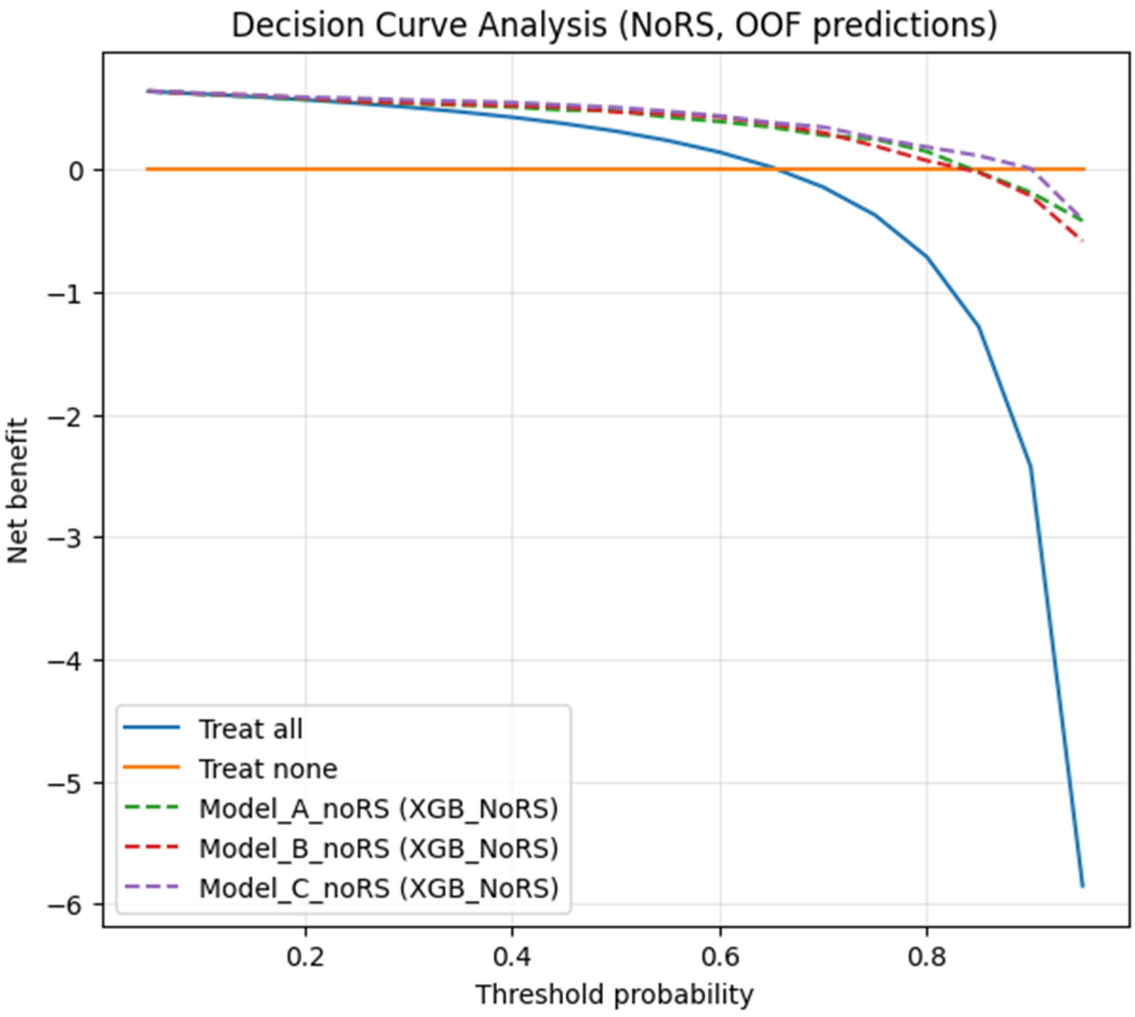

Figure S16. Binned calibration plot based on aligned out-of-fold predicted probabilities for the staged logistic regression models (Model A–C). Points represent grouped prediction bins, showing the relationship between predicted mortality probability and observed event frequency. The jagged appearance reflects the modest sample size and small numbers within some bins.

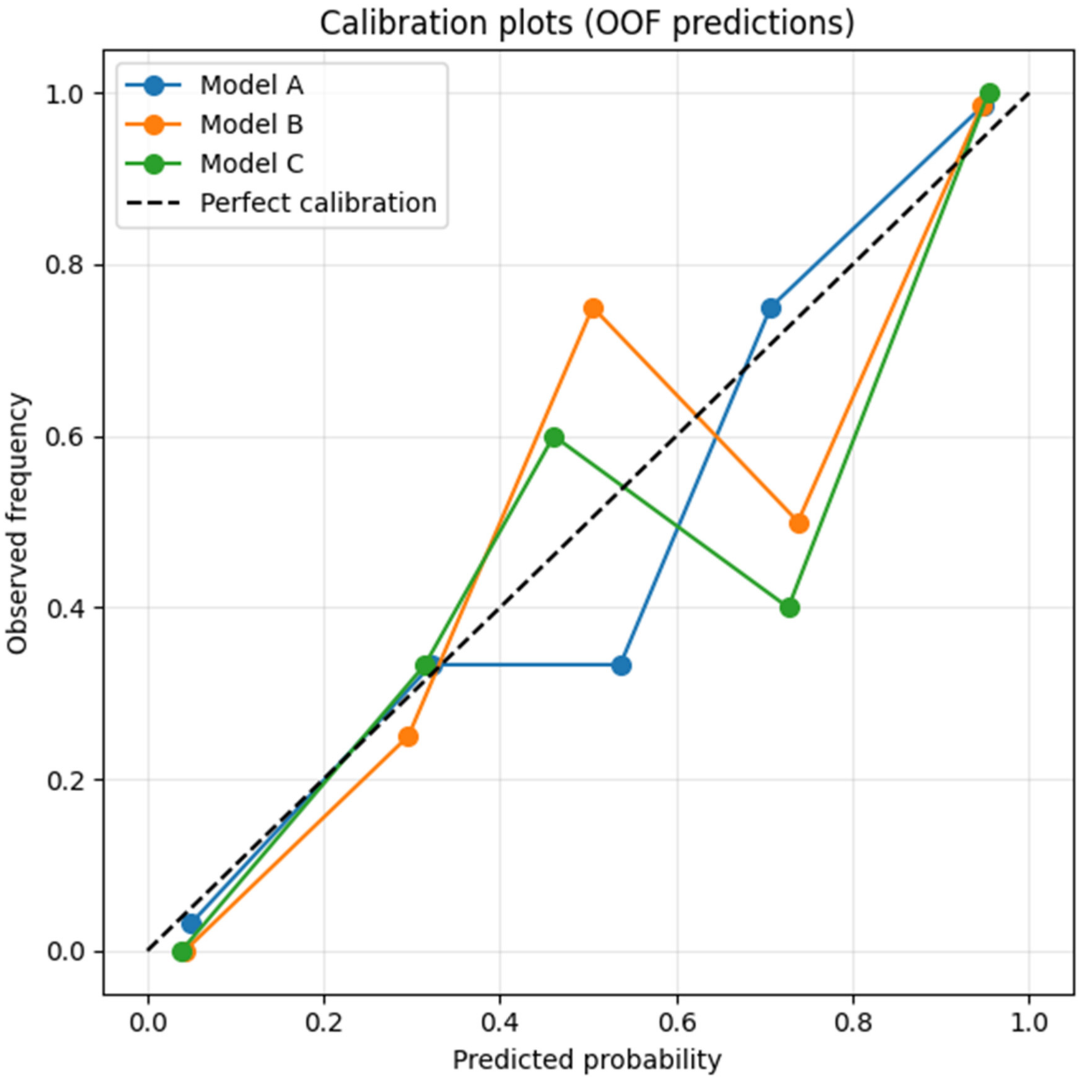

Supplement: Supplementary file 1 [file jcm-15-05056-s001.zip › jcm-4349603-supplementary.pdf]
